# p24–Tango1 interactions ensure ER–Golgi interface stability and efficient transport

**DOI:** 10.1083/jcb.202309045

**Published:** 2024-03-12

**Authors:** Ke Yang, Zhi Feng, José Carlos Pastor-Pareja

**Affiliations:** 1 School of Life Sciences, Tsinghua University, Beijing, China; 2 Tsinghua-Peking Center for Life Sciences, Beijing, China; 3 Institute of Neurosciences, Consejo Superior de Investigaciones Científicas-Universidad Miguel Hernández, San Juan de Alicante, Spain

## Abstract

The eukaryotic p24 family, consisting of α-, β-, γ- and δ-p24 subfamilies, has long been known to be involved in regulating secretion. Despite increasing interest in these proteins, fundamental questions remain about their role. Here, we systematically investigated *Drosophila* p24 proteins. We discovered that members of all four p24 subfamilies are required for general secretion and that their localizations between ER exit site (ERES) and Golgi are interdependent in an α→βδ→γ sequence. We also found that localization of p24 proteins and ERES determinant Tango1 requires interaction through their respective GOLD and SH3 lumenal domains, with Tango1 loss sending p24 proteins to the plasma membrane and vice versa. Finally, we show that p24 loss expands the COPII zone at ERES and increases the number of ER–Golgi vesicles, supporting a restrictive role of p24 proteins on vesicle budding for efficient transport. Our results reveal Tango1–p24 interplay as central to the generation of a stable ER–Golgi interface.

## Teaser

Yang et al. systematically analyze in *Drosophila* the function of the four p24 protein subfamilies and discover that interaction with Tango1 is essential for their concentration between ER and Golgi and for efficiency of COPII-mediated general secretory transport.

## Introduction

Efficient trafficking of secretory cargoes from the endoplasmic reticulum (ER) to the Golgi apparatus is essential for the physiological health and the correct organization of eukaryotic cells. In the eukaryotic secretory pathway, cargoes are collected at specialized regions of the ER called ER exit sites (ERES), from where they are transported to the Golgi with the assistance of the COPII (coat protein complex II) vesicle budding machinery (Bannykh et al., 1996; Barlowe and Miller, 2013; Brandizzi and Barlowe, 2013; Zanetti et al., 2011). In addition to this forward secretory traffic, ERES concentrate as well the income of proteins and membranes that travel in the opposite direction from the Golgi to the ER (Lerich et al., 2012; Roy Chowdhury et al., 2020; Yang et al., 2021). ERES, therefore, are critical traffic junctions mediating both anterograde and retrograde transport. To do this, cells must bring together in the reduced space between ERES and Golgi the numerous cytoplasmic components of the different transport machineries and their multiple regulators. The question of how cells organize and maintain a dynamic but stable ER–Golgi interface for efficient transport in the face of constant forward and reverse membrane traffic has sparked great interest among cell biologists.

**Figure 1.**
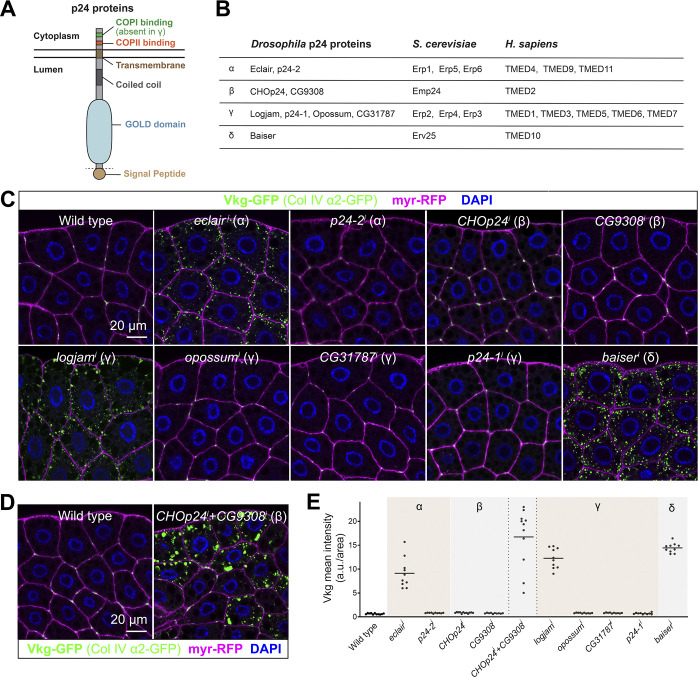
**α-, β-, γ-, and δ-p24 proteins are required for Collagen IV secretion in *Drosophila*. (A)** Schematic domain organization of p24 family proteins, representing their cleavable secretion signal peptide, lumenal GOLD domain, lumenal coiled-coil region, transmembrane region, and short cytoplasmic tail containing COPII/COPI recruitment motifs. **(B)** Classification into α, β, γ, and δ subfamilies of p24 proteins in the fruit fly (*D. melanogaster*), baker’s yeast (*Saccharomyces cerevisiae*), and humans (*Homo sapiens*). **(C and D)** Confocal images of L3 fat body adipocytes from wild-type larvae and larvae where p24-encoding genes have been knocked down under control of fat body driver *BM-40-SPARC-GAL4* individually (C, *BM-40-SPARC>eclair*^*i*^, *>p24-2*^*i*^, *>CHOp24*^*i*^, *>CG9308*^*i*^, *>logjam*^*i*^, *>opossum*^*i*^, *>CG31787*^*i*^, *>p24-1*^*i*^, and *>baiser*^*i*^) and, for β-p24 proteins, in combination (D, *BM-40-SPARC>CHOp24*^*i*^*+CG9308*^*i*^), showing localization of Collagen IV (α2 chain Vkg-GFP in green). Plasma membrane labeled with GAL4-driven myr-RFP (magenta). Nuclei stained with DAPI (blue). **(E)** Quantification of intracellular Collagen IV retention measured from images like those in C and D. Each dot represents a measurement in one cell (*n* ≥ 10 per group). Horizontal lines indicate mean values. See also Fig. S1.

p24 proteins are a family of type-I transmembrane proteins highly conserved among eukaryotes. Identified as major constituents of both COPI (coat protein complex I) and COPII vesicles (Otte et al., 2001; Schimmöller et al., 1995; Sohn et al., 1996; Stamnes et al., 1995), they are long known to be involved in secretion (Kaiser, 2000; Pastor-Cantizano et al., 2016). Based on sequence homology, p24 proteins are classified into four subfamilies: α-, β-, γ- and δ-p24 (Dominguez et al., 1998; Pastor-Cantizano et al., 2016; Strating et al., 2009). p24 proteins of all four subfamilies display a common modular structure (Fig. 1 A) consisting of a cleavable signal peptide, a lumenal part with a Golgi dynamics (GOLD) domain (Anantharaman and Aravind, 2002) and a coiled-coil region involved in oligomerization (Ciufo and Boyd, 2000; Emery et al., 2000), a single hydrophobic transmembrane region, and a short cytosolic tail that contains well-characterized COPI and COPII recruiting motifs responsible for their cycling between the ER and Golgi (Dominguez et al., 1998; Fiedler et al., 1996). Given the striking conservation of their four subfamilies, abundant presence at the ER–Golgi interface, and multiple disease connections (Roberts and Satpute-Krishnan, 2023), understanding the role of p24 proteins has been a prominent research goal in the secretion field for over two decades. However, despite a large number of studies and spiking interest in recent years, fundamental questions about them remain largely unresolved.

p24 proteins are widely believed to function as specific cargo-interacting receptors for a collection of different protein cargoes. These include glycosylphosphatidylinositol (GPI)-anchored proteins (Bernat-Silvestre et al., 2020; Bonnon et al., 2010; Castillon et al., 2011; Manzano-Lopez et al., 2015; Muñiz et al., 2000; Takida et al., 2008), Wnt family ligands (Buechling et al., 2011; Port et al., 2011), G-protein coupled receptors (Luo et al., 2007), fibronectins (Hou and Jerome-Majewska, 2018), plant myrosinase-associated protein GLL23 (Jancowski et al., 2014), insulin (Hosaka et al., 2007; Zhang and Volchuk, 2010), Rac-GAP chimaerin (Wang and Kazanietz, 2002), Toll-like receptor 4 (Liaunardy-Jopeace et al., 2014), and, more recently, leaderless cargoes such as interleukin-1 (Zhang et al., 2020). Furthermore, defective function of p24 proteins is associated with Alzheimer’s disease as mediators of amyloid precursor protein trafficking modulating γ-secretase cleavage (Chen et al., 2006; Hasegawa et al., 2010; Vetrivel et al., 2007). Nevertheless, along with reports of defects in the transport of specific cargoes, evidence of more general impairments upon p24 deficiencies exists in the literature as well, such as altered Golgi–ER retrograde transport (Aguilera-Romero et al., 2008; Gommel et al., 2001; Majoul et al., 1998; Montesinos et al., 2014) and abnormal Golgi morphology (D’Arcangelo et al., 2015; Denzel et al., 2000; Koegler et al., 2010; Lavoie et al., 1999; Mitrovic et al., 2008; Pastor-Cantizano et al., 2018; Rojo et al., 2000). Moreover, broad roles have been ascribed to p24 proteins in mediating ER retention for quality control (Belden and Barlowe, 2001; Dvela-Levitt et al., 2019; Gomez-Navarro et al., 2020; Lopez et al., 2020; Ma et al., 2017; Springer et al., 2000; Wen and Greenwald, 1999), membrane contact during autophagosome formation from the ERGIC (ER Golgi intermediate compartment) (Li et al., 2022), and lipid transfer between the ER and Golgi (Anwar et al., 2022). Fitting all these proposed functions, cargo-specific and general, into a consistent view is problematic. Furthermore, p24 proteins have been reported to function in heteromeric complexes (Füllekrug et al., 1999; Marzioch et al., 1999) and the wide conservation of the four subfamilies hints at important, non-redundant roles in the secretory pathway; however, because of their similar organization and structure, even when members of different subfamilies are compared, the question of whether they play differentiated or redundant roles remains unanswered. Complicating analysis of these issues through genetics, yeast p24 mutants are viable and show only mild defects, even when combined into an octuple mutant where all members of the four p24 subfamilies are deleted (Springer et al., 2000).

The genome of the fruit fly *Drosophila melanogaster* encodes nine proteins of the p24 family, distributed among the four conserved subfamilies as follows (Fig. 1 B): Eclair and p24-2 belong to the α-p24 subfamily; CHOp24 and CG9308 to the β-p24 subfamily; Logjam, CG31787, Opossum, and p24-1 are γ-24 subfamily members; and, finally, Baiser is the only δ-p24 subfamily representative (Carney and Bowen, 2004). In contrast to the situation in yeast, *Drosophila* p24 proteins play clearly essential roles, ubiquitous knockdown of each of them in all tissues producing lethality or severely reduced viability (Saleem et al., 2012). Phenotypic loss of function analysis of *Drosophila* p24 proteins has revealed defects in embryonic patterning (Bartoszewski et al., 2004), oviposition (Boltz et al., 2007; Carney and Taylor, 2003), fecundity and male fertility (Saleem et al., 2012), and stress response (Boltz and Carney, 2008). In addition, detailed mechanistic studies concluded that p24 proteins interact with Wingless and other WNT family ligands and are required for their secretion (Buechling et al., 2011; Port et al., 2011; Zang et al., 2015), raising again the question of whether p24 proteins act in the early secretory pathway as specific receptors for the transport of particular cargoes. Furthermore, regarding the redundancy and relations among the different subfamilies, a systematic analysis of protein localization and mutual functional requirements has not been carried out.

*Drosophila* is a powerful model for investigating protein secretion and the early secretory pathway. Genetic screens in flies have identified conserved new secretory genes (Bard et al., 2006; Ke et al., 2018; Kondylis et al., 2011; Tiwari et al., 2015; Wendler et al., 2010). An additional advantage of researching secretion in *Drosophila* is the availability of sophisticated tools for transgenic tagging and tissue-specific functional interrogation. Many recent studies have taken advantage of these to dissect secretory traffic in an animals (Fujii et al., 2020; Glashauser et al., 2023; Johnson et al., 2020; Ma et al., 2020; Park et al., 2022; Song et al., 2022; van Leeuwen et al., 2018; Zajac and Horne-Badovinac, 2022; Zhou et al., 2023). In *Drosophila*, the early secretory pathway is organized into secretory units, tens to hundreds per cell, in which ERES lie in close proximity to Golgi ministacks (Kondylis and Rabouille, 2009). We have previously characterized the organization of these ERES–Golgi units using 3D-SIM (structured illumination microscopy), TEM (transmission electron microscopy), and FIB-SEM (focused ion beam–SEM) (Yang et al., 2021). Besides occasional continuities between ERES and pre-cis-Golgi, we could distinguish two populations of vesicles at the ER–Golgi interface: one at the center of the ERES cup, corresponding to the highest COPII concentration, and the other in the periphery, consistent in size and localization with retrograde COPI vesicles (Yang et al., 2021). A critical protein in the maintenance of this ER–Golgi interface is Tango1 (transport and Golgi organization 1), an ERES-localized transmembrane protein discovered in a screening in *Drosophila* S2 cells (Bard et al., 2006; Saito et al., 2009). Tango1 is the single *Drosophila* member of the MIA/cTAGE family, only present in animals (Feng et al., 2021). Loss of Tango1 function has been shown to impair secretion of multiple cargoes in all examined *Drosophila* tissues (Lerner et al., 2013; Liu et al., 2017; Pastor-Pareja and Xu, 2011; Reynolds et al., 2019; Ríos-Barrera et al., 2017; Zhang et al., 2014). In absence of Tango1, ERES become smaller and detach from Golgi (Liu et al., 2017), indicating that Tango1, among other roles, can function as a tether and organizer of the ER–Golgi interface (Feng et al., 2021; McCaughey et al., 2021; Saito and Maeda, 2019). The cytoplasmic part of Tango1, capable of self-interacting (Liu et al., 2017), may have a chief role in this organizing function, whereas the role of the ER lumenal part of Tango1, which contains an SH3 domain reported to bind cargoes directly or through adaptors (Arnolds and Stoll, 2023; Ishikawa et al., 2016; Saito et al., 2009; Yuan et al., 2018), is less understood. Mechanisms that ensure concentration of Tango1 at ERES could be of prime importance to regulate their size and protect the stability of the ERES–Golgi interface.

Here, using the larval fat body as a screening system, we have carried out a systematic analysis of the p24 family in *Drosophila.* We show that the presence of members of all four p24 subfamilies is necessary for general secretion and dissect their mutual requirements for localization between ERES and pre-cis-Golgi. We also show that p24 proteins and Tango1 interact in the ER lumen and mutually depend on each other for their localization at the ER–Golgi interface. Finally, our high-resolution analysis through FIB-SEM shows an excess of vesicles in p24 loss conditions. Overall, our results evidence that p24 proteins confer stability to the ER–Golgi interface by limiting COPII budding and preventing Tango1 escape from ERES.

## Results

### All four p24 subfamilies are required for general secretion in *Drosophila*

**Figure S1.**
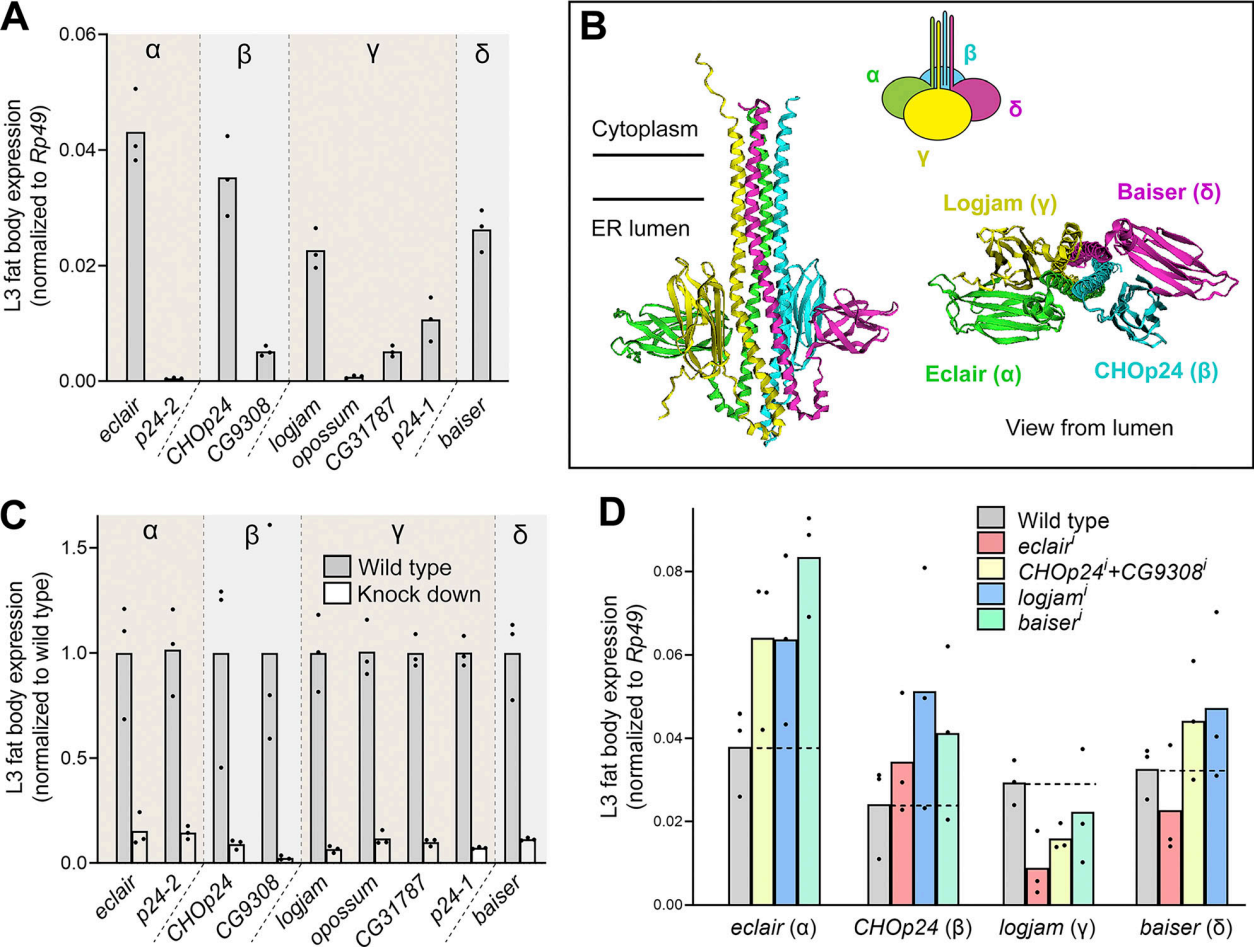
**p24 expression levels in larval fat body cells. (A)** Expression of genes encoding p24 family proteins in L3 fat body adipocytes from wild-type larvae. Expression levels are normalized to that of *Rp49.* **(B)** Prediction of the structure of a putative heterotetramer consisting of the p24 subfamily members most highly expressed in the L3 fat body using AlphaFold2-multimer (https://colab.research.google.com/github/sokrypton/ColabFold/blob/main/AlphaFold2.ipynb). **(C)** Expression of genes encoding p24 family proteins in L3 fat body adipocytes from wild-type larvae and larvae where p24-encoding genes have been knocked down under control of fat body driver *BM-40-SPARC-GAL4* (*BM-40-SPARC* > *eclair*^*i*^, *>p24-2*^*i*^, *>CHOp24*^*i*^, *>CG9308*^*i*^, *>logjam*^*i*^, *>opossum*^*i*^, *>CG31787*^*i*^, *>p24-1*^*i*^, and *>baiser*^*i*^). Expression levels are normalized to wild type. **(D)** Expression level relative to *Rp49* of α-p24 eclair, β-p24 CHOp24, γ-p24 logjam, and δ-p24 baiser in L3 fat body adipocytes from wild-type larvae (gray) and larvae where genes encoding α-p24 Eclair, β-p24 CHOp24+CG9308, γ-p24 Logjam, and δ-p24 Baiser have been knocked down (red, α-p24 *eclair*^*i*^; yellow, β-p24 *CHOp24*^*i*^*+CG9308*^*i*^; blue, γ-p24 *logjam*^*i*^; green, δ-p24 *baiser*^*i*^), all driven by *BM-40-SPARC-GAL4*. The horizontal dashed line marks the expression level in the wild type. Bar heights indicate mean value. Each dot represents a biological replicate (*n* = 3 in each group). Related to Fig. 1.

In a previous screening, we found that *logjam*, encoding a *Drosophila* γ-p24 protein, is required for Collagen IV secretion by fat body adipocytes of the third instar larva (L3 stage) (Ke et al., 2018), the main source of Collagen IV for the basement membranes of the *Drosophila* larva (Pastor-Pareja and Xu, 2011). Examining the expression of all p24 genes through quantitative RT-PCR (qRT-PCR), we found that *eclair* (α), *CHOp24* (β), and *baiser* (δ) were highly expressed in the fat body, similar to *logjam* (γ) (Fig. S1, A and B). To better understand the role of Logjam and p24 proteins in the secretory pathway, we knocked down the expression of the remaining *Drosophila* p24 proteins in the fat body under control of the GAL4-UAS expression system (Fig. S1, C and D) and found that, similar to *logjam* (*logjam*^*i*^), knockdown of *eclair* (*eclair*^*i*^) and *baiser* (*baiser*^*i*^), respectively, encoding α- and δ-p24 subfamily members, led to intracellular retention of Viking-GFP (Vkg-GFP), a functional GFP-trap fusion of the Collagen IV α2 chain (Fig. 1, C and E). While no defect was observed upon single knockdown of *CHOp24* or *CG9308*, encoding the two *Drosophila* β-p24 subfamily members, their simultaneous knockdown (*CHOp24*^*i*^+*CG9308*^*i*^) led to Vkg-GFP intracellular retention (Fig. 1, D and E), proving their intra-subfamily redundancy in the fat body. These results, altogether, show that the functions of members of the α-, β-, γ-, and δ-p24 subfamilies are required in fat body adipocytes for efficient Collagen IV secretion.

**Figure 2.**
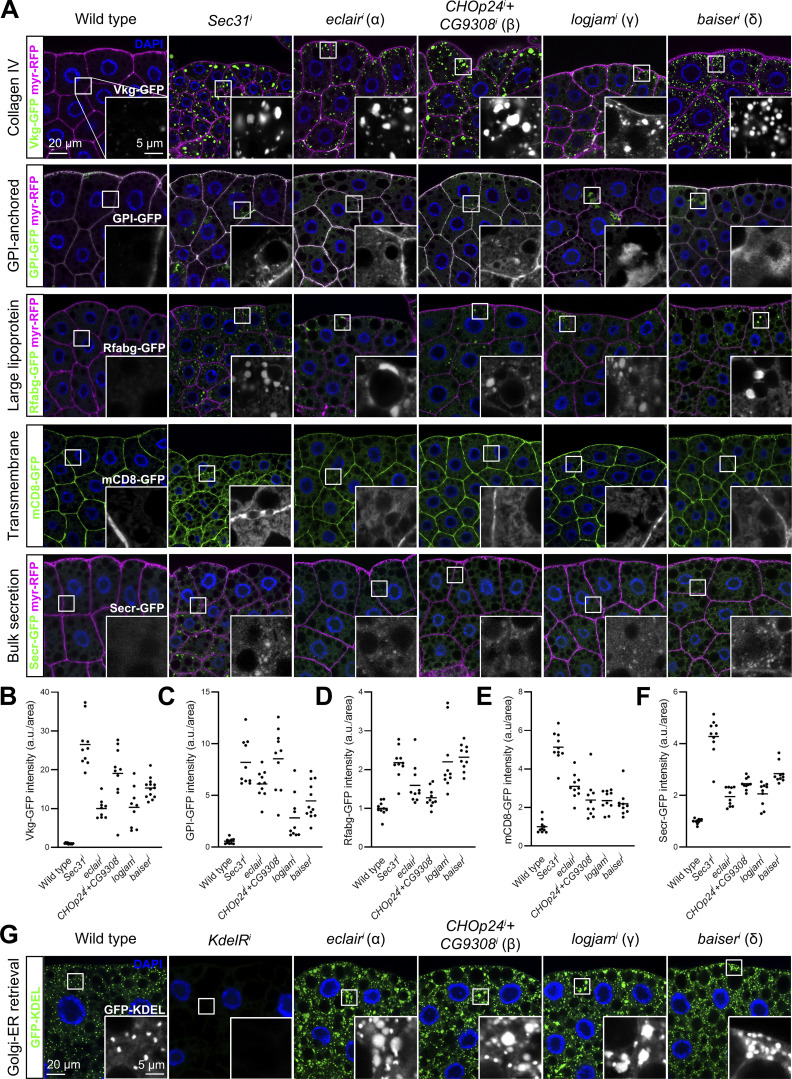
**p24 proteins are required for general ER–Golgi transport. (A)** Confocal images of L3 fat body adipocytes showing localization in green of Vkg-GFP (Collagen IV protein trap), GPI-GFP (driven by *Cg-GAL4*), Rfabg-GFP (driven by endogenous promoter), mCD8-GFP (driven by *BM-40-SPARC-GAL4*), and Secreted-GFP (driven by *BM-40-SPARC-GAL4*). Fat body was dissected from wild-type larvae and larvae where genes encoding COPII coat component Sec31, α-p24 Eclair, β-p24 CHOp24 and CG9308, and γ-p24 Logjam and δ-p24 Baiser had been knocked down under control of fat body drivers *Cg-GAL4* (for GPI-GFP) or *BM-40-SPARC-GAL4* (for Vkg-GFP, Rfabg-GFP, mCD8-GFP, and Secr-GFP). Plasma membrane labeled with GAL4-driven myr-RFP (magenta), except for mCD8-GFP images. Nuclei stained with DAPI (blue). Magnified insets in the lower right corner of each image show isolated GFP signal in white. **(B–F)** Quantification of intracellular retention of Vkg-GFP (B), GPI-GFP (C), Rfabg-GFP (D), mCD8-GFP (E), and Secr-GFP (F), measured from images like those in A. Each dot represents a measurement in one cell (*n* ≥ 10 per group). Horizontal lines indicate mean values. **(G)** Confocal images of L3 fat body adipocytes showing localization in green of GFP-KDEL (driven by *Cg-GAL4*). Fat body was dissected from wild-type larvae and larvae where genes encoding KdelR, Eclair, CHOp24+CG9308, Logjam, and Baiser have been knocked down under control of *Cg-GAL4*. Nuclei stained with DAPI (blue). Magnified insets in the lower right corner of each image show isolated GFP signal in white.

p24 proteins are proposed to function as specific cargo receptors for certain kinds of proteins such as GPI-anchored proteins (Bonnon et al., 2010; Muñiz et al., 2000; Takida et al., 2008) or leaderless cargoes (Zhang et al., 2020). Having shown their requirement in Collagen IV secretion, we decided to test their requirement in the transport of other cargoes. We found that knockdown in fat body adipocytes of *eclair* (α), *CHOp24+CG9308* (β), *logjam* (γ), or *baiser* (δ) caused defective secretion of not just Collagen IV (Fig. 2, A and B) but also of GPI-anchored GFP (GFP fused to GPI attachment signal from CD58) (Fig. 2, A and C), apolipoprotein B-related Rfabg (Fig. 2, A and D), transmembrane protein CD8 (Fig. 2, A and E), and soluble secretion marker secreted GFP (GFP coupled to a signal peptide) (Fig. 2, A and F). Hence, similar to knockdown of COPII coat component Sec31 (Fig. 2, A–F), knockdown of p24 proteins caused defective secretion of all examined cargoes. We additionally examined retrograde transport marker GFP-KDEL, which concentrates at fat body ERES as a result of Golgi-to-ER recycling by the retrograde KDEL receptor (KdelR) (Yang et al., 2021). In contrast with its clearance from the cell upon *KdelR* knockdown (*KdelR*^*i*^), GFP-KDEL showed strong intracellular retention in the ER when we knocked down *eclair* (α), *CHOp24*+*CG9308* (β), *logjam* (γ), or *baiser* (δ) (Fig. 2 G), indicating a primary defect in ER-to-Golgi cargo trafficking. Based on these data, we conclude that p24 proteins of all four subfamilies are required in fat body adipocytes for efficient anterograde transport in the general secretory pathway.

### *Drosophila* p24 proteins concentrate between ERES and pre*-*cis-Golgi

**Figure 3.**
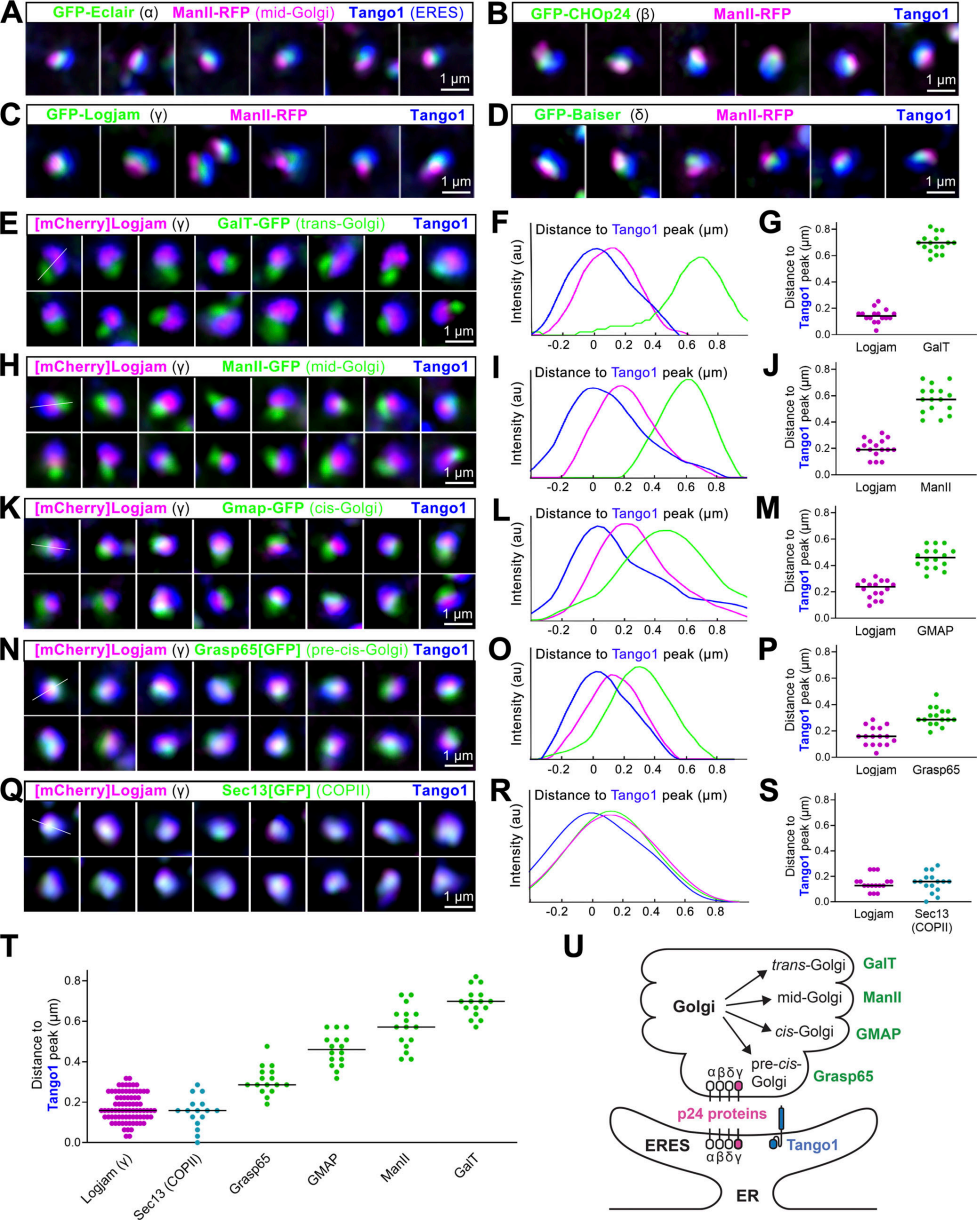
**p24 proteins localize between ERES and pre-cis-Golgi. (A–D)** Superresolution 3D-SIM images of ERES–Golgi units from L3 fat body adipocytes showing localization in green of GFP-tagged α-p24 Eclair (A), β-p24 CHOp24 (B), β-p24 Logjam (C), or δ-p24 Baiser (D), all driven by *Cg-GAL4*, mid-Golgi marker ManII (driven by *Cg-GAL4*, magenta), and ERES marker Tango1 (anti-Tango1, blue). **(E, H, K, N, and Q)** Superresolution 3D-SIM images of ERES–Golgi units from L3 fat body adipocytes showing localization of endogenous γ-p24 Logjam ([mCherry]Logjam CRISPR/Cas9 knock-in, magenta) in relation to ERES Tango1 (anti-Tango1, blue) and, in green, trans-Golgi GalT-GFP (E, driven by *Cg-GAL4*), mid-Golgi ManII-GFP (H, driven by *Cg-GAL4*), cis-Golgi GMAP-GFP (K, protein trap), pre-cis-Golgi Grasp65[GFP] (N, CRISPR/Cas9 knock-in), and COPII coatomer Sec13[GFP] (Q, CRISPR/Cas9 knock-in). Images are maximum intensity projections of three to five sections (A–D, E, H, K, N, and Q). **(F, I, L, O, and R)** Signal profiles across individual ERES–Golgi units following the white lines in the upper left images in E, H, K, N, and Q, respectively. **(G, J, M, P, S, and T)** Graphs representing peak distances with respect to Tango1 in signal profiles like those in F, I, L, O, and R, respectively. The horizontal lines indicate mean values. Each dot represents a measurement in one ERES–Golgi unit profile (G, J, M, P, S, *n* = 16 per group). Results summarized in T. **(U)** Schematic depiction of the localization of p24 proteins within an ERES–Golgi unit, as deduced from 3D-SIM image analysis.

To better understand the role of p24 proteins in the *Drosophila* secretory pathway, we next investigated their localization. To visualize p24 proteins, we added a GFP tag to the N-terminal of Eclair (α), CHOp24 (β), Logjam (γ), and Baiser (δ) after their signal peptides and expressed these tagged forms in fat body adipocytes. Through super-resolution 3D-SIM, we observed that p24 proteins localized at ERES–Golgi units, concentrating between the ERES (marker Tango1) and Golgi (mid-Golgi marker Mannosidase II) (Fig. 3, A–D). To confirm this, we created transgenic flies in which we knocked in an mCherry tag at the N-terminal of Logjam after its signal peptide using CRISPR/Cas9 technology and used this endogenous [mCherry]Logjam to study in detail its localization within ERES–Golgi units. To do this, we imaged Logjam and ERES marker Tango1 together with markers of different Golgi compartments (Yang et al., 2021): trans-Golgi marker GalT (Fig. 3, E–G), mid-Golgi marker ManII (Fig. 3, H–J), cis-Golgi marker GMAP (Fig. 3, K–M), and pre*-*cis-Golgi marker Grasp65 (Fig. 3, N–P; CRISPR/Cas9 Grasp65[GFP] knock-in). As evidenced by signal plot profiles and peak distance quantification, Logjam localization is distinct from those of GalT, ManII, and GMAP, while its highest concentration is closer to pre-cis-Golgi Grasp65 (Fig. 3 l). Furthermore, of all examined markers, γ-p24 Logjam most closely resembled COPII coatomer Sec13 (Fig. 3, Q–T; CRISPR/Cas9 Sec13[GFP] knock-in), suggesting a close relation with the COPII vesicle budding machinery. Our data, therefore, place p24 protein localization at the ER–Golgi interface (Fig. 3 U), consistent with cycling between ERES and pre*-*cis-Golgi.

### p24 protein localizations are interdependent in an α→βδ→γ sequence

**Figure 4.**
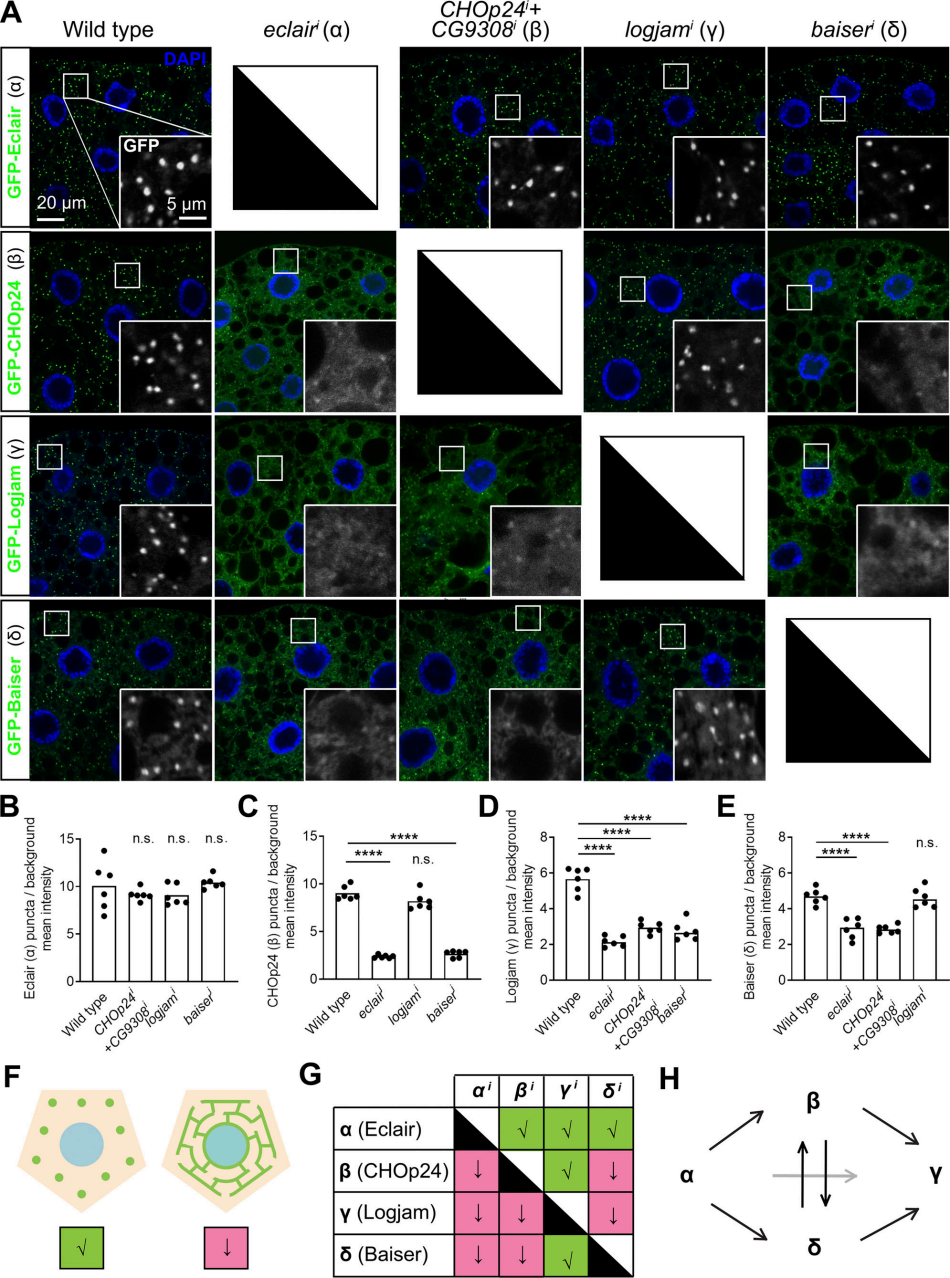
**p24 protein localizations are interdependent in an α→βδ→γ sequence. (A)** Confocal images of L3 fat body adipocytes showing localization in green of GFP-tagged α-p24 Eclair, β-p24 CHOp24, γ-p24 Logjam, and δ-p24 Baiser, all driven by *Cg-GAL4*. Fat body was dissected from wild-type larvae and larvae where genes encoding Eclair, CHOp24+CG9308, Logjam, or Baiser had been knocked down under control of *Cg-GAL4*. Magnified insets in the lower right corner of each image show an isolated GFP signal in white. Nuclei stained with DAPI (blue). **(B–E)** Graphs quantifying the effect on the localization of GFP-tagged Eclair (B), CHOp24 (C), Logjam (D), and Baiser (E) of the knockdown of indicated p24-encoding genes, measured from images like those in A. Graphs represent the ratio between the amounts of GFP signal concentrated in puncta and diffuse signal. Each dot represents a measurement from one cell (*n* = 6 in each group). Bar heights indicate the mean value. P values from Brown-Forsythe ANOVA and Dunnett’s multiple comparisons tests (B, P = 0.7702 [n.s.] for *CHOp24*^*i*^*+CG9308*^*i*^, 0.7700 [n.s.] for *logjam*^*i*^, and 0.9802 [n.s.] for *baiser*^*i*^), and one-way ANOVA and Dunnett’s multiple comparisons tests (C, P < 0.0001 [****] for *eclair*^*i*^, = 0.0735 [n.s.] for *logjam*^*i*^, and <0.0001 [****] for *baiser*^*i*^; D, P < 0.0001 [****] for *eclair*^*i*^, *CHOp24*^*i*^*+CG9308*^*i*^, and *baiser*^*i*^; E, P < 0.0001 [****] for *eclair*^*i*^ and *CHOp24*^*i*^*+CG9308*^*i*^, = 0.9846 [n.s.] for *logjam*^*i*^). **(F)** Illustration of punctate localization (√) and diffuse ER distribution (↓) observed for p24 proteins in A. **(G)** Summary of the effect of the knockdown of indicated p24-encoding genes on the localization of Eclair, CHOp24, Logjam, and Baiser, according to B–E. **(H)** Model depicting requirements among p24 protein subfamilies for correct localization, as deduced from G.

p24 proteins have been reported to form heteromeric complexes (Füllekrug et al., 1999; Marzioch et al., 1999). Our finding that deficiency in each of the four p24 subfamilies resulted in defects in general secretion, and their similar localization at the ER–Golgi interface, led us to explore possible mutual requirements for their localization. To do this, we knocked down in the fat body the expression of *eclair* (α), *CHOp24*+*CG9308* (β), *logjam* (γ), or *baiser* (δ), and examined the effect of their loss in the localization of the remaining. In this way, we found that α-p24 Eclair concentration in ERES–Golgi units was unaffected by the loss of p24 proteins of the other subfamilies (Fig. 4, A and B). In contrast, localization of γ-p24 Logjam was defective when we knocked down the expression of members of each of the three other p24 subfamilies (Fig. 4, A and D), displaying a more diffuse ER distribution (Fig. 4 F). As for β-p24 CHOp24 and δ-p24 Baiser, their correct concentration depended on the presence not only of α-p24 Eclair but also of each other (Fig. 4, A, C, and E). Summarizing all these results together (Fig. 4 G), our analysis revealed an α→βδ→γ hierarchy for the correct localization of p24 proteins. In this hierarchy (Fig. 4 H), α-p24 is first to localize, independently, between ERES and pre-cis-Golgi, β- and δ-p24 are mutually dependent and dependent on the presence of α-p24, and, finally, γ-p24 is unable to concentrate in the absence of any of the other three.

### Localization of p24 proteins depends on a GOLD-SH3 interaction with Tango1

**Figure 5.**
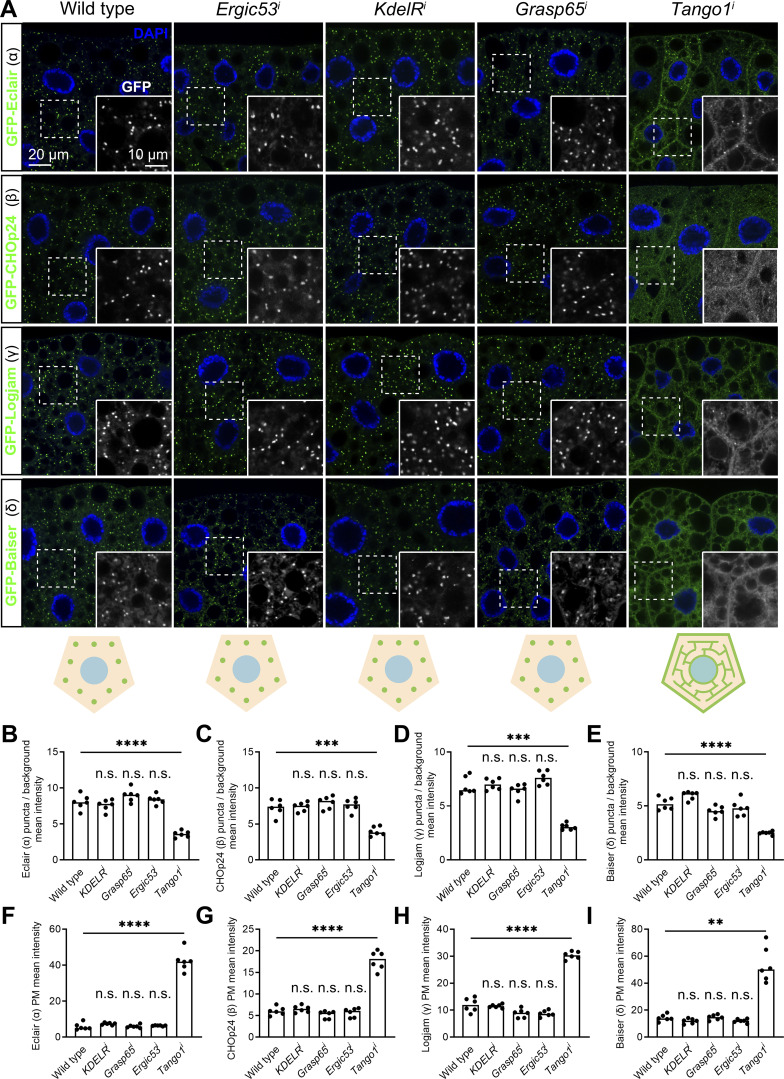
**Concentration of p24 proteins at the ER–Golgi interface depends on Tango1. (A)** Confocal images of L3 fat body adipocytes showing localization in green of GFP-tagged α-p24 Eclair, β-p24 CHOp24, γ-p24 Logjam, and δ-p24 Baiser, all driven by *Cg-GAL4*. Fat body was dissected from wild-type larvae and larvae in which genes encoding Ergic53, KdelR, Grasp65, and Tango1 had been knocked down under control of *Cg-GAL4*. Nuclei stained with DAPI (blue). Magnified insets in the lower right corner of each image show the isolated GFP signal in white. Distribution of p24 proteins illustrated in bottom cartoons. **(B–I)** Graphs quantifying the effect of the knockdown of *KdelR*, *Grasp65*, *Ergic53*, and *Tango1* on the localization of GFP-tagged Eclair (B and F), CHOp24 (C and G), Logjam (D and H), and Baiser (E and I), measured from images like those in A. Graphs represent the ratio between the amounts of puncta and diffuse signal (B–E), and mean intensity in plasma membrane (F–I). Each dot represents a measurement from a cell (*n* = 6 in each group). Bar heights indicate mean values. P values from ordinary one-way ANOVA and Dunnett’s multiple comparisons tests (B, P = 0.7695 [n.s.] for *KDELR*^*i*^, = 0.1470 [n.s.] for *Grasp65*^*i*^, = 0.8484 [n.s.] for *Ergic53*^*i*^, and <0.0001 [****] for *Tango1*^*i*^; E, P = 0.0504 [n.s.] for *KDELR*^*i*^, = 0.0863 [n.s.] for *Grasp65*^*i*^, = 0.4216 [n.s.] for *Ergic53*^*i*^, and <0.0001 [****] for *Tango1*^*i*^; F, P = 0.7108 [n.s.] for *KDELR*^*i*^, = 0.9998 [n.s.] for *Grasp65*^*i*^, = 0.9918 [n.s.] for *Ergic53*^*i*^, and <0.0001 [****] for *Tango1*^*i*^), and Brown-Forsythe ANOVA and Dunnett’s multiple comparisons tests (C, P = 0.9997 [n.s.] for *KDELR*^*i*^, = 0.6949 [n.s.] for *Grasp65*^*i*^, = 0.9645 [n.s.] for *Ergic53*^*i*^, and = 0.008 [***] for *Tango1*^*i*^; D, P = 0.9945 [n.s.] for *KDELR*^*i*^, = 0.8134 [n.s.] for *Grasp65*^*i*^, = 0.3662 [n.s.] for *Ergic53*^*i*^, and = 0.0001 [***] for *Tango1*^*i*^; G, P = 0.8200 [n.s.] for *KDELR*^*i*^, = 0.4532 [n.s.] for *Grasp65*^*i*^, = 0.9568 [n.s.] for *Ergic53*^*i*^, and <0.0001 [****] for *Tango1*^*i*^; H, P = 0.9869 [n.s.] for *KDELR*^*i*^, = 0.1215 [n.s.] for *Grasp65*^*i*^, = 0.0874 [n.s.] for *Ergic53*^*i*^, and <0.0001 [****] for *Tango1*^*i*^; I, P = 0.2888 [n.s.] for *KDELR*^*i*^, = 0.9960 [n.s.] for *Grasp65*^*i*^, = 0.3076 [n.s.] for *Ergic53*^*i*^, and = 0.0025 [**] for *Tango1*^*i*^). See also Fig. S2.

**Figure S2.**
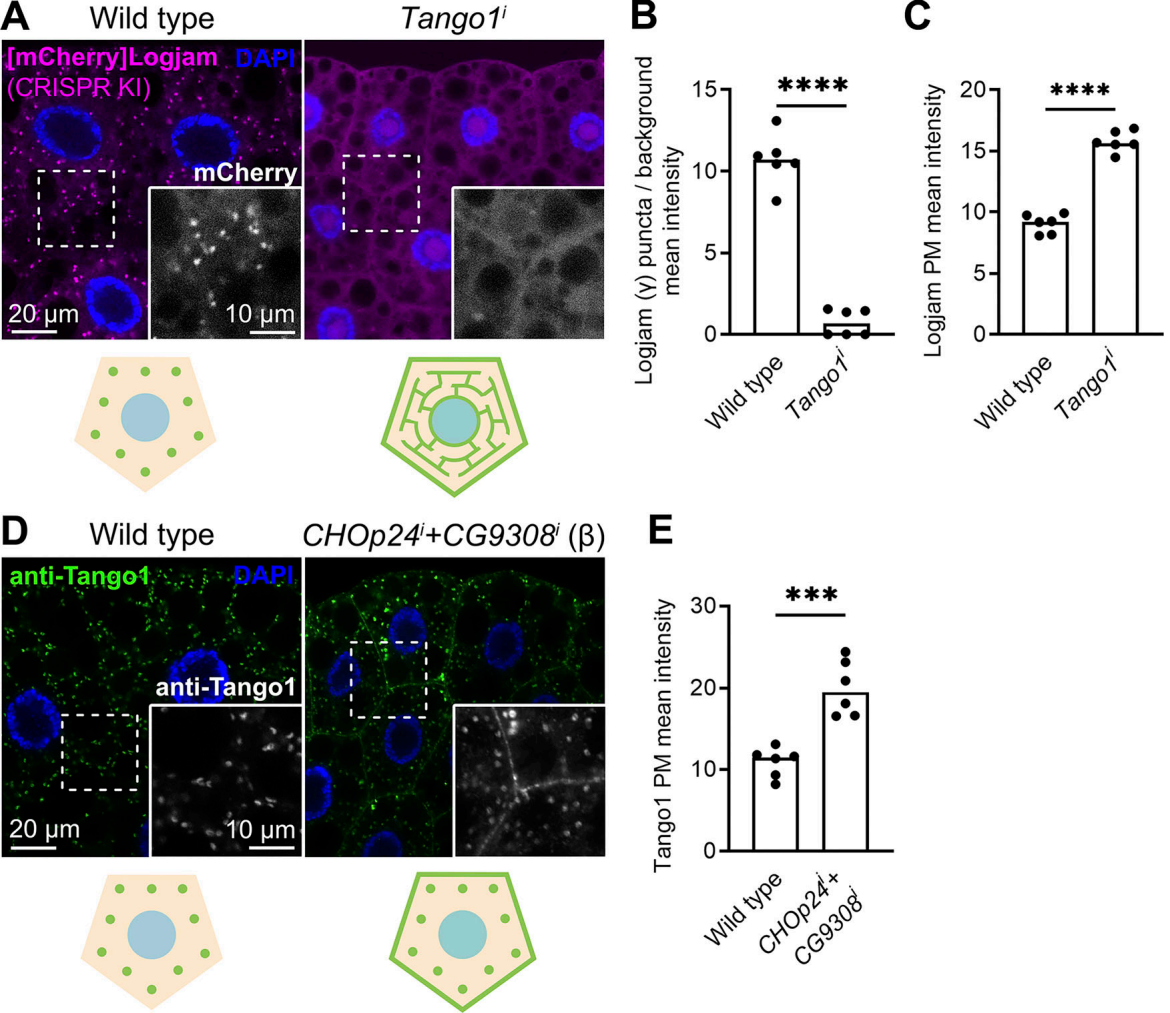
**Tango1–p24 localizations are mutually dependent. (A)** Confocal images of L3 fat body adipocytes showing localization of endogenous γ-p24 Logjam ([mCherry]Logjam CRISPR/Cas9 knock-in, magenta). Fat body was dissected from wild-type larvae and larvae in which the gene encoding Tango1 had been knocked down under control of *Cg-GAL4*. Magnified insets in the lower right corner of each image show the isolated Logjam signal in white. **(B and C)** Graphs quantifying the effect of *Tango1* knockdown on the intracellular (B) and plasma membrane (C) localization of Logjam, measured from images like those in A. Graph represents the ratio between the amounts of puncta and diffuse signal (B), and mean intensity of plasma membrane (PM) signal (C). **(D)** Confocal images of L3 fat body adipocytes showing localization of endogenous Tango1 (anti-Tango1, green). Fat body was dissected from wild-type larvae and larvae where genes encoding β-p24 CHOp24 and CG9308 have been knocked down under control of *BM-40-SPARC-GAL4*. Magnified insets in the lower right corner of each image show the isolated Tango1 signal in white. Nuclei stained with DAPI (blue). **(E)** Graphs quantifying the effect on the plasma membrane localization of Tango1 of β-p24 *CHOp24*^*i*^*+CG9308*^*i*^, measured from images like those in D. Graph represents mean intensity of plasma membrane signal. Each dot represents a measurement from one cell (B, C, and E) (*n* = 6 in each group). Bar heights indicate mean value. P values from unpaired *t* tests (B, P < 0.0001 [****]; C, P < 0.0001 [****]; E, P = 0.0002 [***]). Related to Figs. 5 and 7.

After observing dramatic changes in the localization of p24 proteins in our experiments, we proceeded to further investigate how p24 proteins maintain their steady localization at the ER–Golgi interface. To do that, we first knocked down in the fat body the expression of known *Drosophila* anterograde and retrograde ER–Golgi transport receptors encoded by *Ergic53* (*Ergic53*^*i*^) and *KdelR*, respectively, to explore whether they were involved in p24 localization, but found no difference in their normal punctate pattern when we imaged GFP-tagged versions of Eclair (α), CHOp24 (β), Logjam (γ), and Baiser (δ) (Fig. 5). Similarly, we observed no apparent defect in the localization of Eclair (α), CHOp24 (β), Logjam (γ), and Baiser (δ) upon knockdown of *Grasp65* (*Grasp65*^*i*^; Fig. 5), encoding a protein of the pre-cis-Golgi required for secretion (Yang et al., 2021). In contrast to these, the distribution of p24 proteins of all four subfamilies strikingly changed upon knockdown of ERES protein Tango1 (*Tango*^*i*^), showing diffuse ER localization and presence at the plasma membrane (Fig. 5). Endogenous γ-p24 Logjam tagged with mCherry displayed ER and plasma membrane mislocalization in *Tango1*^*i*^ adipocytes as well (Fig. S2, A–C), confirming that Tango1 is required for the correct localization of p24 proteins.

**Figure 6.**
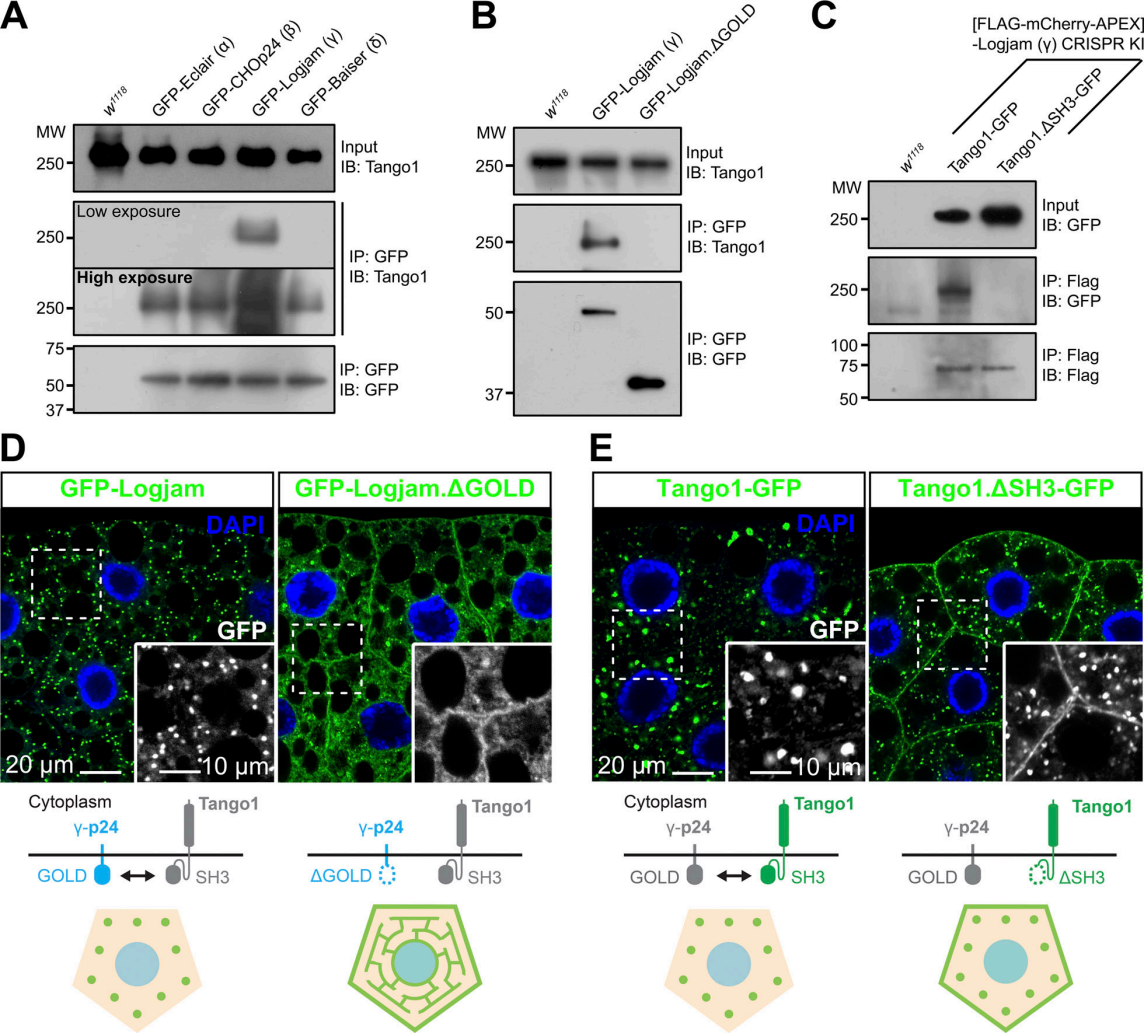
**Tango1 and γ-p24 Logjam interact through their respective SH3 and GOLD domains. (A)** Immunoblot analysis of Tango1–p24 interaction. GFP-tagged α-p24 Eclair, β-p24 CHOp24, γ-p24 Logjam, and δ-p24 Baiser expressed under *Cg-GAL4* control were immunoprecipitated (IP) from L3 fat body lysates and immunoblotted (IB) with anti-Tango1 (both low- and high-exposure images are shown). **(B)** Immunoblot analysis of Tango1–Logjam interaction. Full-length and GOLD domain–deleted Logjam (Logjam.ΔGOLD), both GFP-tagged and expressed under *Cg-GAL4* control, were immunoprecipitated (IP) from L3 fat body lysates and immunoblotted (IB) with anti-Tango1. **(C)** Immunoblot analysis of Tango1–Logjam interaction. [FLAG]Logjam (CRISPR/Cas9 knock-in) was immunoprecipitated (IP) from L3 fat body lysates and immunoblotted (IB) with anti-GFP to detect full-length Tango1 and SH3 domain–deleted Tango1 (Tango1.ΔSH3), both GFP-tagged and expressed under *Cg-GAL4* control. As controls, *w*^*1118*^ fat body was processed in parallel (A–C), and lysates and immunoprecipitates were immunoblotted, respectively, with anti-Tango1 and anti-GFP (A and B) or anti-GFP and anti-Flag (C). Uncropped scans are provided in the source data. **(D and E)** Confocal images of L3 fat body adipocytes showing in green the localization of full-length and GOLD-deleted γ-p24 Logjam (D), and full-length and SH3-deleted Tango1 (E), all GFP-tagged and driven by *Cg-GAL4*. Magnified insets in the lower right corner of each image show an isolated GFP signal in white. Nuclei stained with DAPI (blue). Protein interactions and distribution patterns of Logjam (D) and Tango1 (E) are schematically illustrated at the bottom. See also Fig. S3. Source data are available for this figure: SourceData F6.

**Figure S3.**
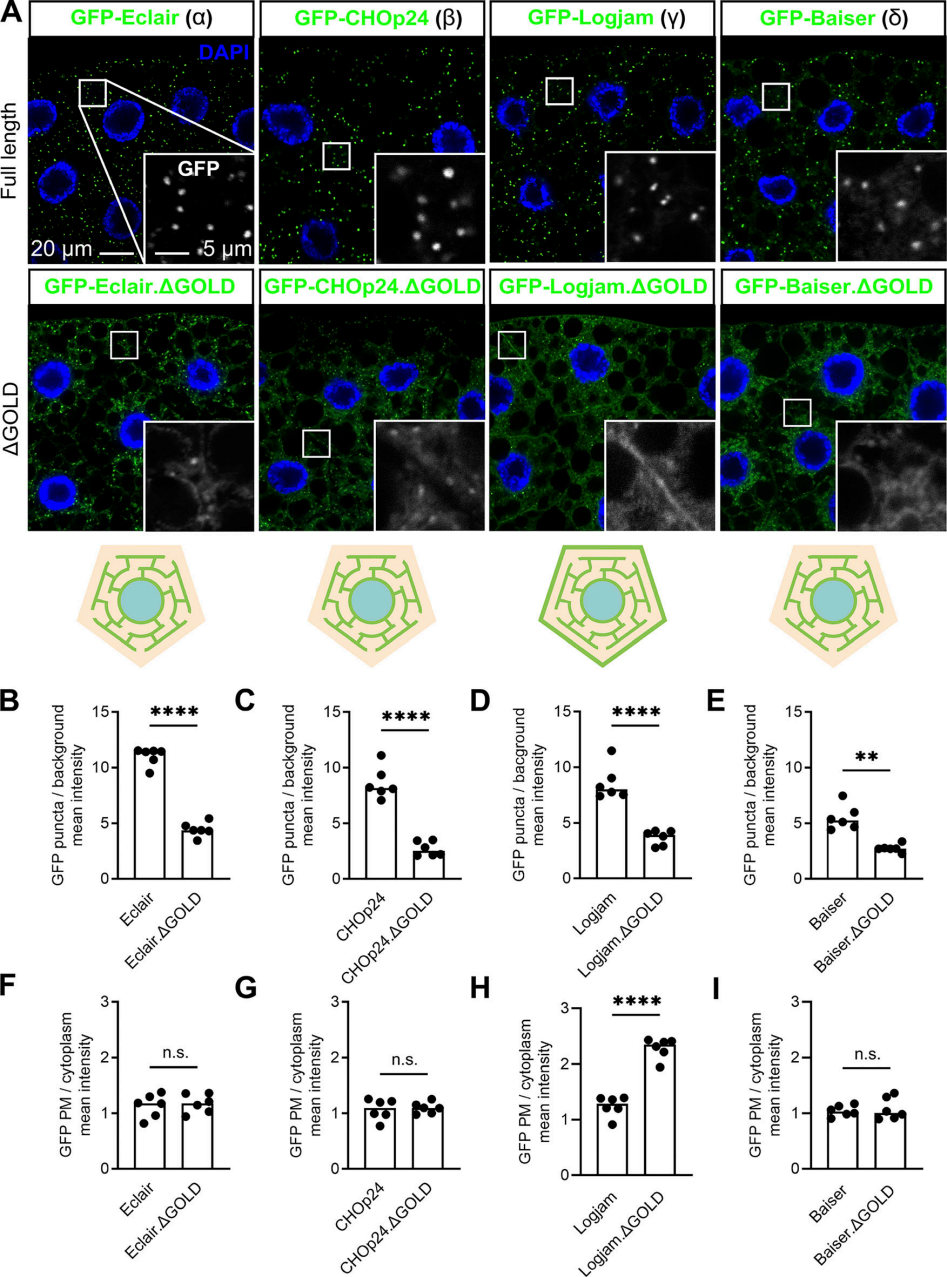
**GOLD domain is required for correct p24 localization. (A)** Confocal images of L3 fat body adipocytes showing localization in green of GFP-tagged full-length α-p24 Eclair, β-p24 CHOp24, γ-p24 Logjam, and δ-p24 Baiser, and corresponding GOLD domain deletion mutants, all driven by *Cg-GAL4*. Nuclei stained with DAPI (blue). Magnified insets in the lower right corner of each image show isolated GFP signal in white. **(B–I)** Graphs quantifying the effect of GOLD deletions on the intracellular (B–E) and plasma membrane (PM) distribution (F–I) of GFP-tagged Eclair (B and F), CHOp24 (C and G), Logjam (D and H), and Baiser (E and I), measured from images like those in A. Graphs represent the ratio between puncta and diffuse signal (B–E) and the mean intensity of plasma membrane signal (F–I). Each dot represents a measurement from one cell (*n* = 6 in each group). Bar heights indicate the mean value. P values from unpaired two-sided *t* tests (B–D, P < 0.0001 [****]; F, P = 0.7354 [n.s.]; G, P = 0.7542 [n.s.]; H, P < 0.0001 [****]; I, P = 0.6757 [n.s.]), and Welch’s *t* test (E, P = 0.0012 [**]). Related to Fig. 6.

Next, we used coimmunoprecipitation followed by western blotting to investigate the possibility that p24 proteins interacted with Tango1, required for their localization. We were, in this way, able to detect Tango1 when we immunoprecipitated GFP-tagged versions of Logjam (γ) and, to a lesser extent, Eclair (α), CHOp24 (β), and Baiser (δ) from fat body adipocytes (Fig. 6 A). Because of the short length of the cytoplasmic tails of the p24 proteins (10 to 14 amino acid residues), we hypothesized that an interaction with Tango1 would most likely involve the ER lumenal part of the protein, where the conserved GOLD domain is found. To test this, we expressed in the fat body a GFP-tagged version of Logjam from which we had deleted its GOLD domain (GFP-Logjam.ΔGOLD) and found that GOLD deletion abolished its interaction with Tango1 (Fig. 6 B). Similarly, deletion of the ER lumenal SH3 domain from a GFP-tagged version of Tango1 (Tango1.ΔSH3-GFP) prevented interaction with endogenous FLAG-tagged Logjam (Fig. 6 C). In addition to these coimmunoprecipitation experiments, we monitored the localization of GFP-Logjam.ΔGOLD and Tango1.ΔSH3-GFP and found in both cases that the truncated proteins failed to localize correctly. In the case of Logjam, GOLD deletion resulted in ER and plasma membrane localization (Fig. 6 D; and Fig. S3, A, D, and H), similar to the effect of Tango1 knockdown (see Fig. 5). Furthermore, besides Logjam, GOLD deletion resulted in mislocalization of Eclair (α), CHOp24 (β), and Baiser (δ) (Fig. S3). Finally, for Tango1, deleting its SH3 domain produced strong presence of the protein in the plasma membrane (Fig. 6 E), suggesting its escape from ERES. Altogether, these results show that Tango1 and p24 proteins interact through their respective SH3 and GOLD lumenal domains, which are required for the correct localization of both.

### Maintenance of Tango1 at the ER–Golgi interface requires p24 proteins

**Figure 7.**
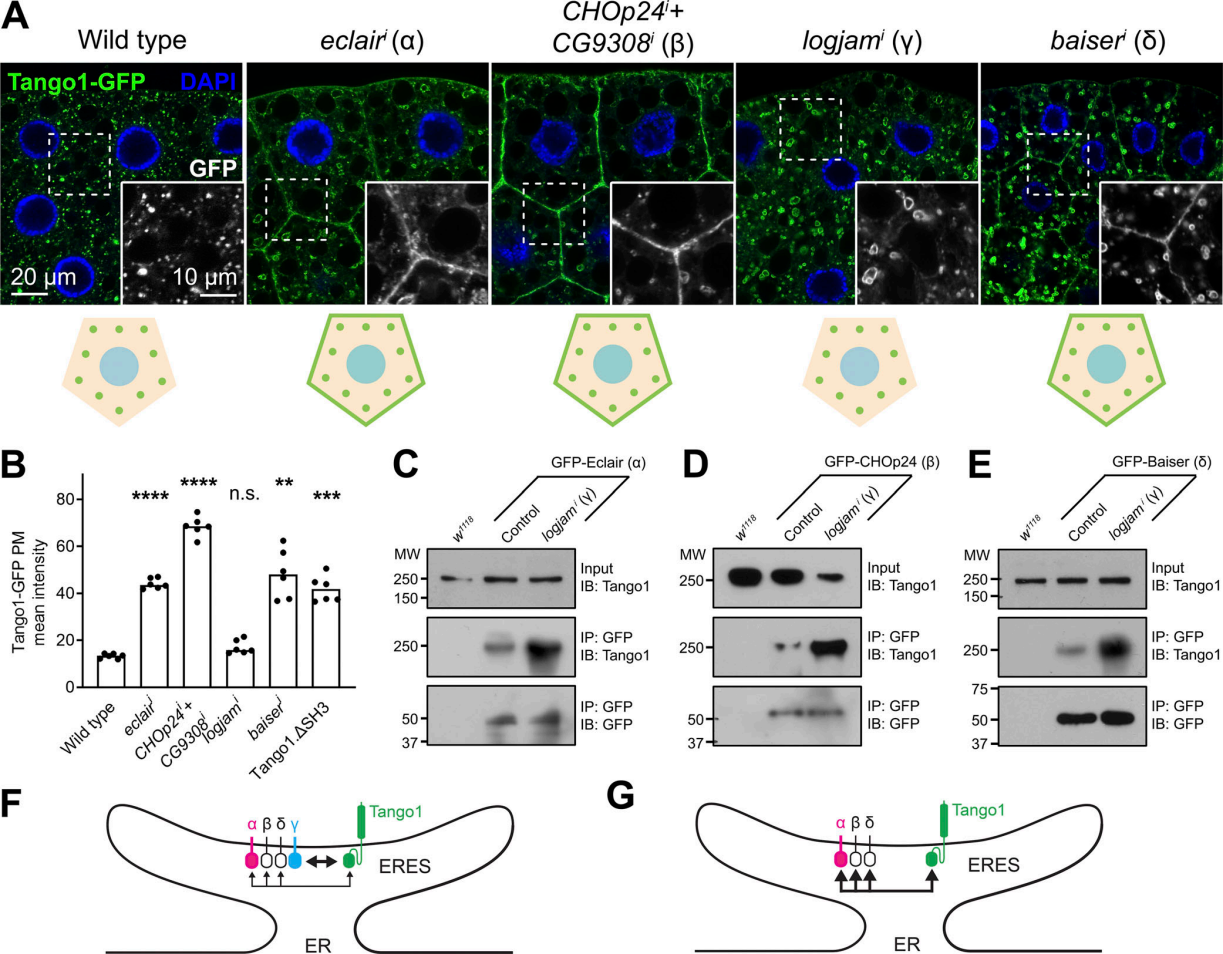
**Loss of α-, β-, or δ- but not γ-p24 causes Tango1 escape to the plasma membrane. (A)** Confocal images of L3 fat body adipocytes showing in green localization of GFP-tagged Tango1 driven by *Cg-GAL4*. Fat body was dissected from wild-type larvae and larvae in which genes encoding α-p24 Eclair, β-p24 CHOp24+CG9308, γ-p24 Logjam, or δ-p24 Baiser had been knocked down. Magnified insets in the lower right corner of each image show the isolated Tango1-GFP signal in white. Tango1 distribution patterns are schematically illustrated at the bottom. Nuclei stained with DAPI (blue). **(B)** Graphs quantifying the effect on the localization of Tango1 of *Cg-GAL4-driven* α-p24 *eclair*^*i*^, β-p24 *CHOp24*^*i*^*+CG9308*^*i*^, γ-p24 *logjam*^*i*^, and δ-p24 *baiser*^*i*^, measured from images like those in A, as well as the effect of SH3 deletion (see Fig. 6 E). Each dot represents a measurement from one cell (*n* = 6 in each group). Bar heights indicate mean value. P values from Brown-Forsythe ANOVA and Dunnett’s multiple comparisons tests (P < 0.0001 [****] for *eclair*^*i*^, <0.0001 [****] for *CHOp24*^*i*^*+CG9308*^*i*^, = 0.0768 [n.s.] for *logjam*^*i*^, = 0.0014 [**] for *baiser*^*i*^, = 0.0003 [***] for Tango1.ΔSH3). **(C–E)** Immunoblot analysis of Tango1–p24 interaction. GFP-tagged α-p24 Eclair (C), β-p24 CHOp24 (D), and δ-p24 Baiser (E), all expressed under *Cg-GAL4* control, were immunoprecipitated (IP) from control and *logjam*^*i*^ L3 fat body lysates and immunoblotted (IB) with anti-Tango1. As additional controls, *w*^*1118*^ fat body was processed in parallel, and lysates and immunoprecipitates were immunoblotted with anti-Tango1 and anti-GFP, respectively. Uncropped scans are provided in the source data. **(F and G)** Schematic illustrations of Tango1–p24 interaction in presence (F) or absence (G) of γ-p24 Logjam. Arrow thickness represents interaction strength. See also Fig. S2. Source data are available for this figure: SourceData F7.

Our experiments, revealing that Tango1 is required for localization of p24 proteins, additionally suggest that the converse is true as well, as hinted by Tango1.ΔSH3 mislocalization. To confirm the requirement of p24 proteins in Tango1 localization, we examined the localization of a GFP-tagged version of Tango1 upon knockdown of p24 proteins. We found that knockdown of *eclair* (α), *CHOp24+CG3908* (β), or *baiser* (δ) resulted in mislocalization of Tango1 to the plasma membrane (Fig. 7 A). Mislocalization of endogenous Tango1 could be detected as well with an antibody (Fig. S2, D and E). Interestingly, however, knockdown of *logjam* (γ) failed to produce this effect (Fig. 7, A and B), suggesting that other p24 proteins, with which Tango1 interacts as well (Fig. 6 A), could compensate for the loss of Logjam to retain Tango1 at ERES. Consistent with this, coimmunoprecipitation experiments showed increased interaction between Tango1 and Eclair (α) (Fig. 7 C), CHOp24 (β) (Fig. 7 D), and Baiser (δ) (Fig. 7 E) when *logjam* expression was knocked down. From these results, we conclude that p24 proteins prevent Tango1 escape from ERES. In addition, our data indicate that in their lumenal interaction with other proteins like Tango1, p24 subfamilies may show some functional redundancy (Fig. 7, F and G), in contrast to their non-redundant, interdependent requirements for localization.

### Loss of p24 proteins expands COPII zone at ERES

**Figure 8.**
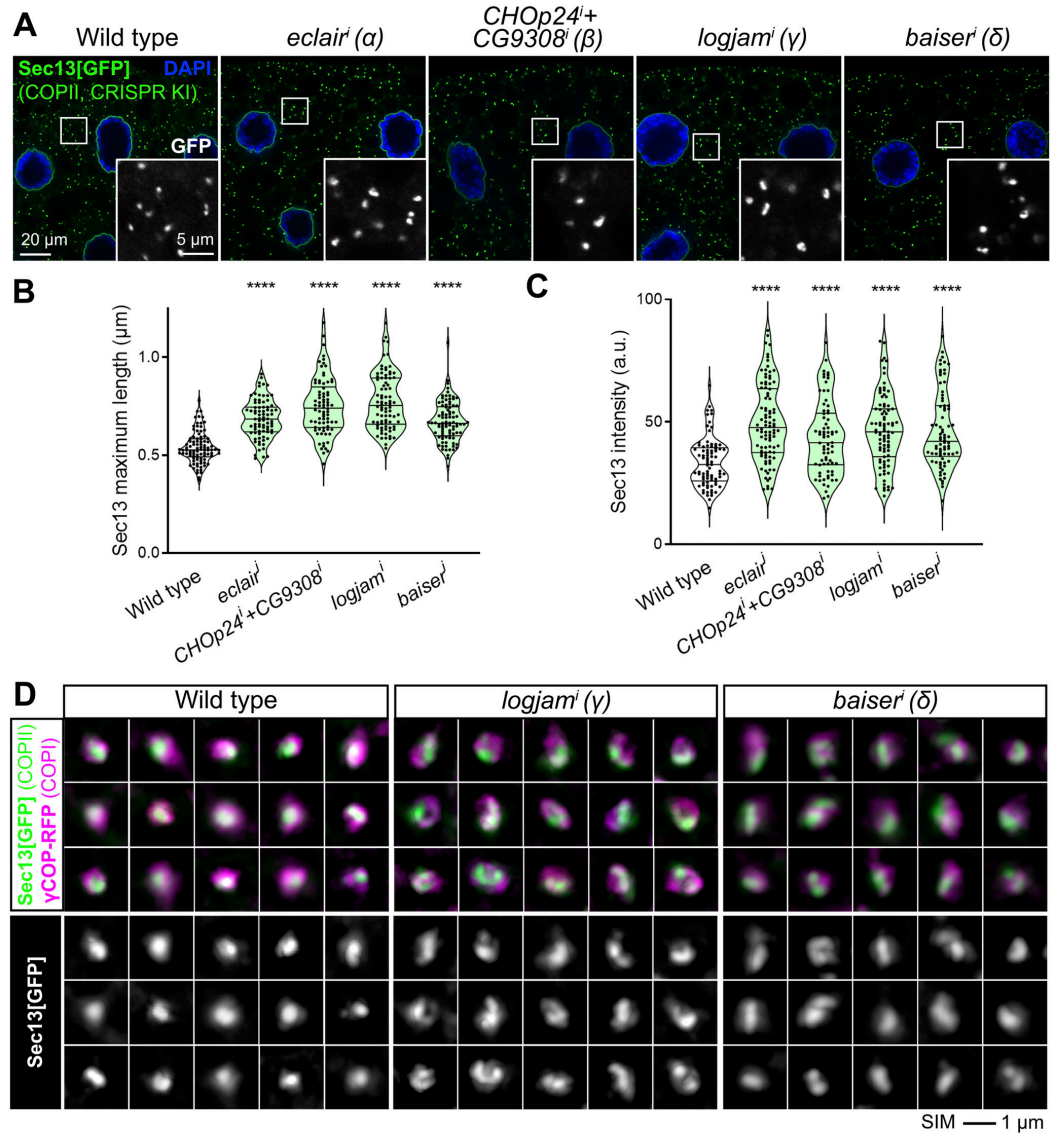
**Loss of p24 increases COPII recruitment and expands COPII zone at ERES. (A)** Confocal images of L3 fat body adipocytes showing in green the localization of COPII coatomer Sec13[GFP] (CRISPR/Cas9 knock-in). Fat body was dissected from wild-type larvae and larvae in which genes encoding α-p24 Eclair, β-p24 CHOp24+CG9308, γ-p24 Logjam, or δ-p24 Baiser had been knocked down under control of *Cg-GAL4*. Magnified insets in the lower right corner of each image show isolated Sec13[GFP] signal in white. Nuclei stained with DAPI (blue). **(B and C)** Quantification of maximum length (B) and intensity (C) of Sec13 puncta measured in images like those in A. Violin plots depict the median value and interquartile range. Each dot represents a measurement in one punctum (*n* > 70 in each group). P values from Brown-Forsythe ANOVA and Dunnett’s multiple comparisons tests (B and C, P < 0.0001 [****] in all cases). **(D)** Superresolution 3D-SIM images of ERES–Golgi units from L3 fat body adipocytes showing localization of Sec13[GFP] (CRISPR/Cas9 knock-in, green) and γCOP-RFP (driven by *Cg-GAL4*, magenta). Fat body was dissected from wild-type, *logjam*^*i*^, and *baiser*^*i*^ larvae (*Cg-GAL4*–driven knockdown). Bottom images show the isolated Sec13[GFP] signal in white. Images are maximum intensity projections of three to five sections. See also Fig. S4.

**Figure S4.**
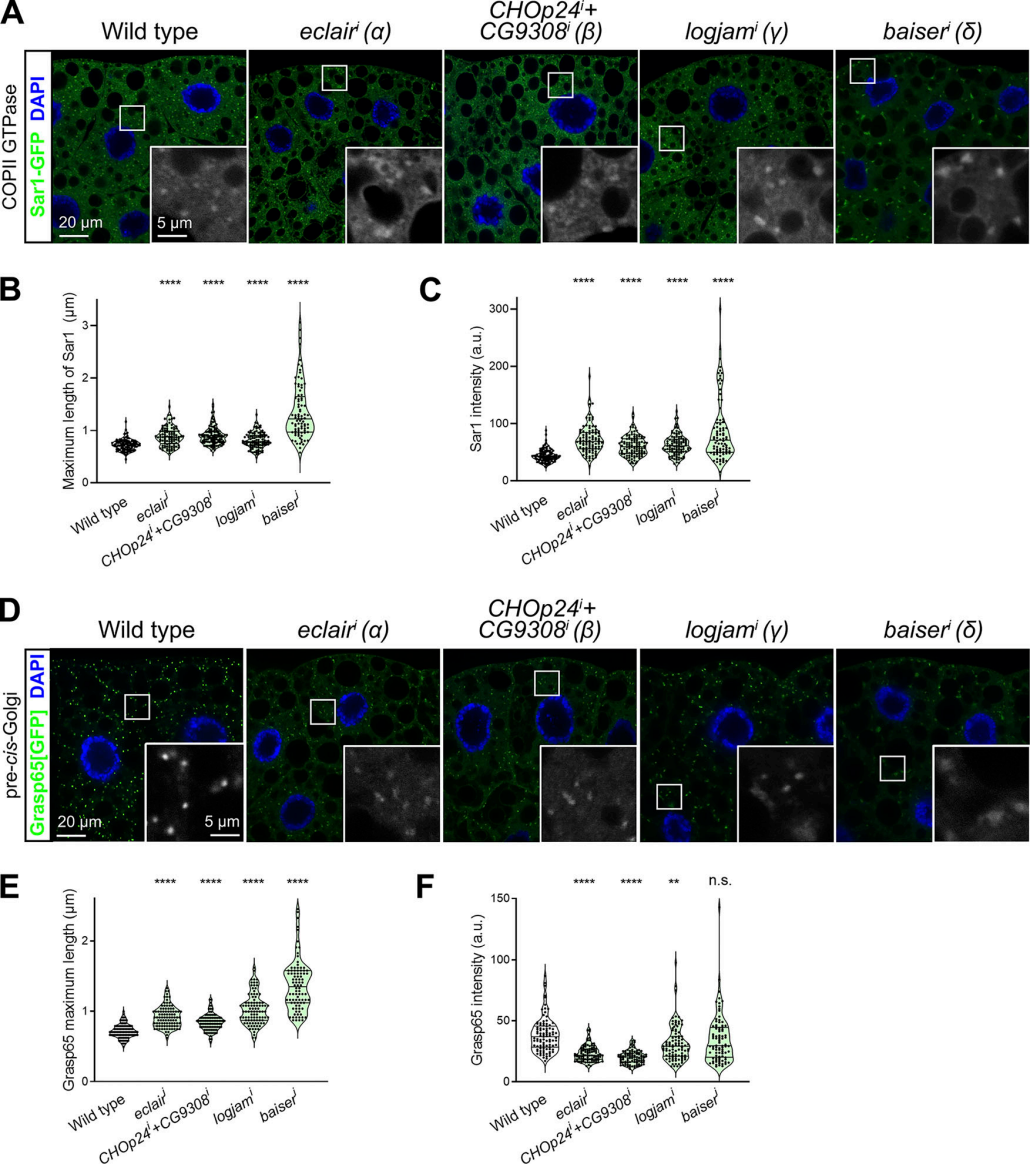
**p24 loss increases Sar1 recruitment and enlarges pre-cis-Golgi. (A and D)** Confocal images of L3 fat body adipocytes showing in green localization of COPII GTPase Sar1-GFP (driven by Cg-GAL4, A) and Grasp65[GFP] (CRISPR/Cas9 knock-in, D). Fat body was dissected from wild-type larvae and larvae in which genes encoding α-p24 Eclair, β-p24 CHOp24 and CG9308, γ-p24 Logjam, or δ-p24 Baiser had been knocked down under control of *Cg-GAL4*. Magnified insets in the lower right corner of each image show an isolated Sec13[GFP] signal in white. Nuclei stained with DAPI (blue). **(B, C, E, and F)** Quantification of maximum length (B and E) and intensity (C and F) of Sar1 puncta (B and C; measured in images like those in A) and Grasp65 puncta (E and F; measured in images like those in D). Violin plots depict median value and interquartile range. P values from Brown-Forsythe ANOVA and Dunnett’s multiple comparisons tests (B, C, and E, P < 0.0001 [****] for *eclair*^*i*^, *CHOp24*^*i*^*+CG9308*^*i*^, *logjam*^*i*^, and *baiser*^*i*^; F, P < 0.0001 [****] for *eclair*^*i*^ and *CHOp24*^*i*^*+CG9308*^*i*^, = 0.0027 [**] for *logjam*^*i*^, and = 0.4924 [n.s.] for *baiser*^*i*^). Related to Fig. 8.

To better understand the role of p24 proteins, and given their colocalization with COPII (Fig. 3, Q–T), we imaged endogenous GFP-tagged Sec13 (Sec13[GFP] knock-in) in the fat body upon knockdown of *eclair* (α), *CHOp24*+*CG9308* (β), *logjam* (γ), or *baiser* (δ) (Fig. 8 A). In all four cases, Sec13 puncta in ERES–Golgi units exhibited a significant increase in their size and intensity (Fig. 8, B and C). Similarly, we could also detect an increase in the size and intensity of puncta formed by the COPII GTPase Sar1 (Fig. S4, A–C) and an enlargement of puncta positive for pre-cis-Golgi marker Grasp65 (Fig. S4, D–F), suggesting an expansion of this Golgi compartment. We have previously shown that in *Drosophila* ERES–Golgi units, COPII concentrates in the center of ERES cups whereas COPI displays a complementary localization around COPII in the ERES periphery (Yang et al., 2021). To further characterize the alteration in COPII caused by the absence of p24 function, we used 3D-SIM to image simultaneously COPII coat component Sec13 and COPI coat component γCOP. When we knocked down *logjam* (γ) or *baiser* (δ), in contrast with the concentration of COPII contained to the center of wild-type ERES, Sec13 signal expanded, partially overlapping peripheral COPI and adopting cup/doughnut morphologies typical of the latter (Fig. 8 D). Overall, these results demonstrate that p24 loss leads to an expansion of the COPII zone at ERES, strongly suggesting that p24 proteins serve an antagonistic role with respect to the COPII budding machinery.

### p24 proteins prevent excess vesicle budding

**Figure S5.**
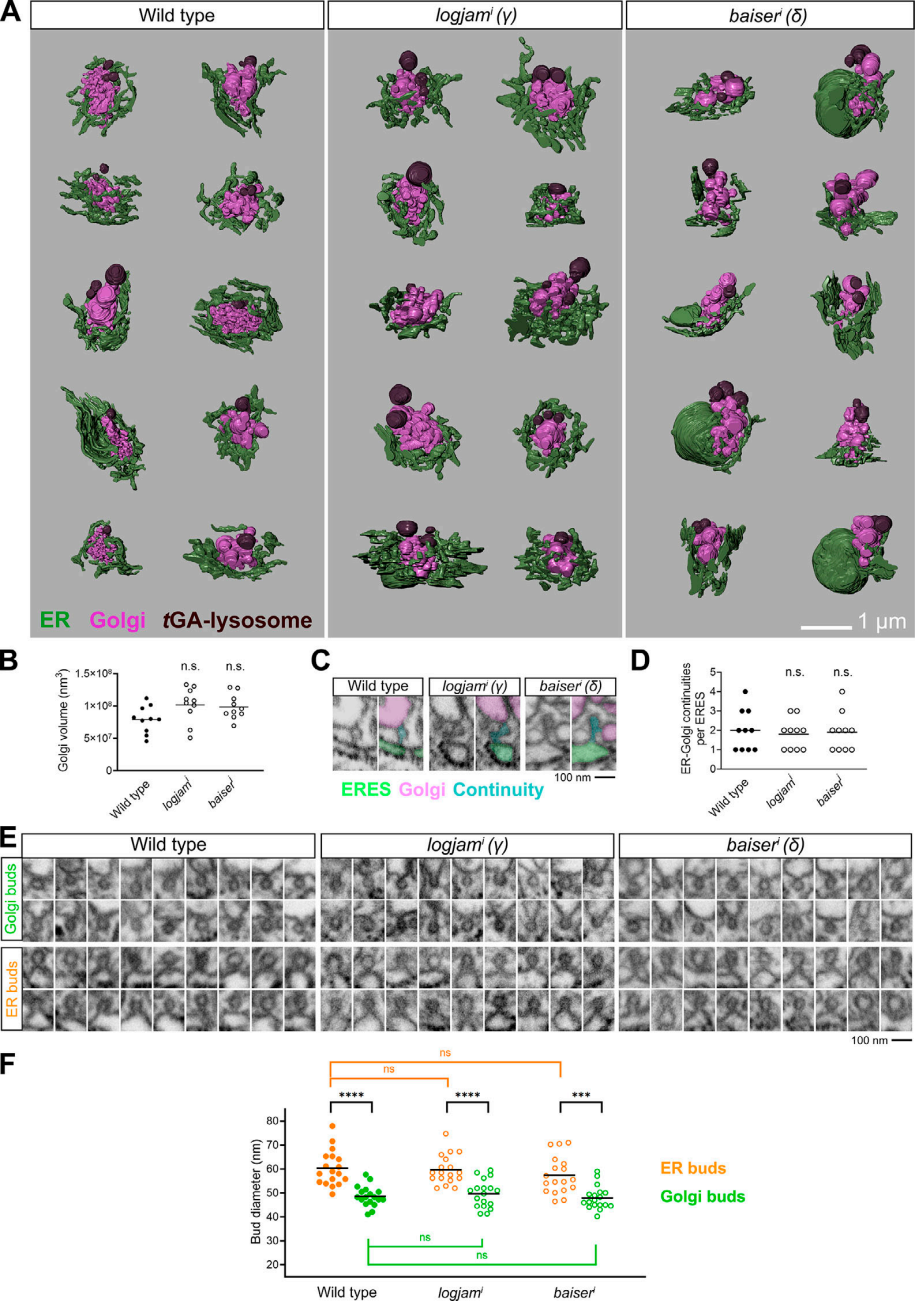
**FIB-SEM analysis of mutant p24 ERES–Golgi units. (A)** 3D reconstructions of ERES–Golgi units from FIB-SEM images of wild-type, *logjam*^*i*^, and *baiser*^*i*^ L3 fat body adipocytes (knockdown driven by *BM-40-SPARC-GAL4*). 10 ERES–Golgi units per genotype were reconstructed for our FIB-SEM analysis. Different colors indicate ER (green), Golgi (pink), and trans-Golgi associated (tGA) lysosomes (Zhou et al., 2023) (brown). **(B)** Golgi volume in wild-type, *logjam*^*i*^, and *baiser*^*i*^ ERES–Golgi units. **(C)** FIB-SEM images of ERES–Golgi continuities in wild-type, *logjam*^*i*^, and *baiser*^*i*^ ERES–Golgi units. Different colors in the color-coded version of each image indicate ERES (green), Golgi (pink), and continuity (cyan). **(D)** Number of ERES–Golgi continuities in wild-type, *logjam*^*i*^, and *baiser*^*i*^ ERES–Golgi units. Each dot represents an ERES–Golgi unit (B and D, *n* = 10 in each group). **(E)** FIB-SEM images exemplifying vesicle buds found in ERES and pre-cis-Golgi in wild-type, *logjam*^*i*^, and *baiser*^*i*^ ERES–Golgi units. **(F)** Quantification of the apparent diameter of ER (orange) and Golgi (green) buds in wild-type, *logjam*^*i*^, and *baiser*^*i*^ ERES–Golgi units. Each dot represents one bud (*n* = 18 in each group). Horizontal lines indicate mean value (B, D, and F). P values from one-way ANOVA and Dunnett’s multiple comparisons tests (B, P = 0.0622 [n.s.] for *logjam*^*i*^ and 0.1162 [n.s.] for *baiser*^*i*^; D, P = 0.8636 [n.s.] for *logjam*^*i*^ and 0.9605 [n.s.] for *baiser*^*i*^; F, P = 0.9655 [n.s.] for *logjam*^*i*^ ER buds, 0.5630 [n.s.] for *baiser*^*i*^ ER buds, 0.7925 [n.s.] for *logjam*^*i*^ Golgi buds, and 0.9905 [n.s.] for *baiser*^*i*^ Golgi buds), two-sided Welch’s *t* test (F, P < 0.0001 [****] for ER versus. Golgi buds in wild type, = 0.0002 [***] for ER versus Golgi buds in *baiser*^*i*^), and unpaired two-sided *t* test (F, P < 0.0001 [****] for ER versus Golgi buds in *logjam*^*i*^). Related to Fig. 9.

**Figure 9.**
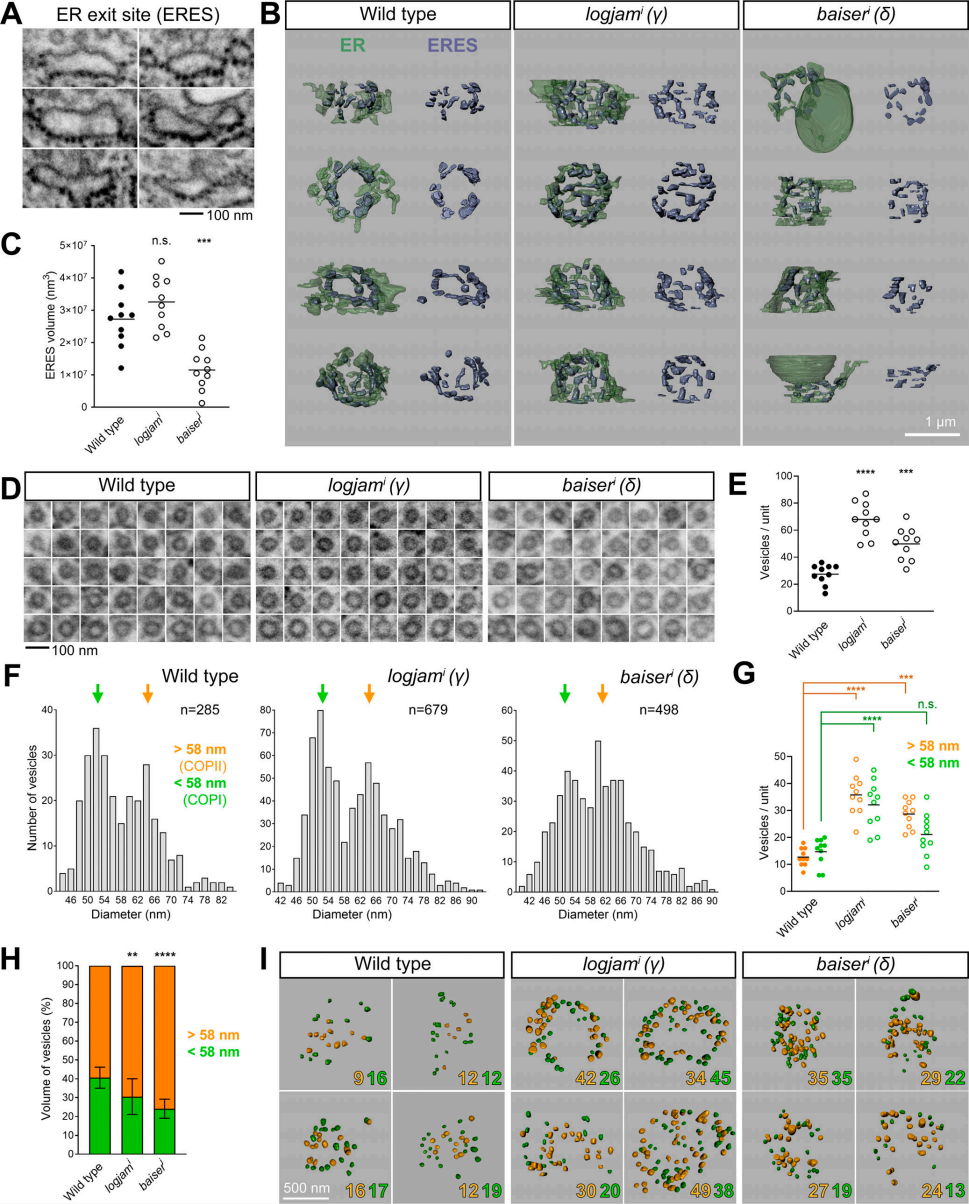
**FIB-SEM analysis reveals ERES size reduction and increased vesicle budding upon p24 loss. (A)** FIB-SEM images featuring examples of ERES areas, devoid of ribosomes on their Golgi-facing side. **(B)** 3D reconstructions of ERES cups from FIB-SEM images of wild-type, *logjam*^*i*^, and *baiser*^*i*^ L3 fat body adipocytes (knockdown driven by *BM-40-SPARC-GAL4*). Proper ERES (purple) are shown separately from ER (green) on the right side. **(C)** ERES volume in wild-type, *logjam*^*i*^, and *baiser*^*i*^ ERES–Golgi units. **(D)** FIB-SEM images exemplifying vesicles found between ERES and Golgi in wild-type, *logjam*^*i*^, and *baiser*^*i*^ ERES–Golgi units. **(E)** Number of vesicles in wild-type, *logjam*^*i*^, and *baiser*^*i*^ ERES–Golgi units. **(F)** Frequency distribution of apparent vesicle diameters in wild-type, *logjam*^*i*^, and *baiser*^*i*^ ERES–Golgi units. Arrows indicate approximate peaks at 52 and 64 nm. **(G)** Number of vesicles larger (orange) and smaller (green) than a 58-nm-diameter threshold in wild-type, *logjam*^*i*^, and *baiser*^*i*^ ERES–Golgi units. Horizontal lines indicate mean value, with each dot representing one ERES–Golgi unit (C, E, and G, *n* = 10 in each group). **(H)** Percentage of added vesicle volume corresponding to vesicles larger (orange) and smaller (green) than 58 nm in wild-type, *logjam*^*i*^, and *baiser*^*i*^ ERES–Golgi units. Data represented as mean ± SD (*n* = 10 in each group). **(I)** Spatial distribution of vesicles larger (orange) and smaller (green) than 58 nm in wild-type, *logjam*^*i*^, and *baiser*^*i*^ ERES–Golgi units. Counts for each annotated in the bottom right corner of 3D reconstructions. The plane of view in reconstructions is perpendicular to the cis–trans axis ERES–Golgi units (B and I). P values from one-way ANOVA and Dunnett’s multiple comparisons tests (C, P = 0.2282 [n.s.] for *logjam*^*i*^ and 0.0002 [***] for *baiser*^*i*^; E, P < 0.0001 [****] for *logjam*^*i*^ and = 0.0002 [***] for *baiser*^*i*^; G, P < 0.0001 [****] for *logjam*^*i*^ > 58 nm, = 0.0002 [***] for *baiser*^*i*^ > 58 nm, <0.0001 for *logjam*^*i*^ < 58 nm, and = 0.1102 [n.s.] for *baiser*^*i*^ < 58 nm; H, P = 0.0063 [**] for *logjam*^*i*^ and <0.0001 [****] for *baiser*^*i*^). See also Fig. S5.

Intrigued by the observed expansion of COPII, we decided to further characterize the effect of p24 loss using FIB-SEM. To do this, we imaged with 20-nm z resolution volumes of wild-type, *logjam*^i^ (γ), and *baiser*^*i*^ (δ) fat body (two samples per genotype) and 3D-reconstructed ERES–Golgi units within them (10 units per genotype; Fig. S5 A). ERES, recognizable as regions of Golgi-facing ER devoid of ribosomes (Fig. 9 A), were reduced in size upon knockdown of *baiser* (δ), but not *logjam* (γ) (Fig. 9, B and C). This is consistent with our earlier finding that Tango1 escapes ERES upon knockdown of *baiser*, but not *logjam* (Fig. 7 A). Meanwhile, Golgi volume did not significantly change compared with the wild type (Fig. S5 B). We next analyzed tubular continuities we had previously discovered between ERES and pre-cis-Golgi (Yang et al., 2021), similar to ERES–ERGIC tubes others have independently described in cultured human cells (Shomron et al., 2021; Weigel et al., 2021), but found no difference in their frequency (around two per unit) across the three genotypes (Fig. S5, C and D). Besides tubular continuities, we identified between ERES and Golgi abundant vesicles in all three genotypes (Fig. 9 D). The number of these vesicles, however, showed a greater than twofold increase in *logjam*^i^ (γ) and *baiser*^*i*^ (δ) conditions compared with the wild type (Fig. 9 E). In the distribution of their sizes, vesicles from wild type, *logjam*^i^ (γ), and *baiser*^*i*^ (δ) alike displayed a two-peaked diameter distribution, with peaks located at 52 and 64 nm (Fig. 9 F), consistent with COPI and COPII vesicle populations, respectively (Yang et al., 2021). When we separately analyzed vesicles by their diameter with a cutoff at 58 nm, the number of vesicles >58 nm increased in both *logjam*^i^ (γ) and *baiser*^*i*^ (δ) ERES–Golgi units (Fig. 9 G). Furthermore, the added volume of >58-nm vesicles increased with respect to <58-nm vesicles in both *logjam*^i^ (γ) and *baiser*^*i*^ (δ) (Fig. 9 H). We also analyzed the diameter of vesicular buds growing from ERES and Golgi (Fig. S5 E). In wild-type ERES–Golgi units, same as in *logjam*^i^ (γ) and *baiser*^*i*^ (δ), ERES buds were larger than Golgi buds, further supporting the existence of two populations of COPII and COPI vesicles at the ERES–Golgi interface in all three genotypes; at the same time, neither ERES nor Golgi buds significantly varied in diameter among the three genotypes (Fig. S5 F), indicating that p24 loss did not change their size. Finally, when we mapped the position of vesicles within the ERES cup, we observed that in *logjam*^i^ (γ) and *baiser*^*i*^ (δ) more >58-nm vesicles were found in a now crowded peripheral zone (Fig. 9 I), reminiscent of our 3D-SIM data documenting COPII expansion (Fig. 8 D). In summary, our FIB-SEM analysis revealed a decrease in ERES size upon *baiser* (δ) knockdown and an increase in the number of vesicles between ERES and pre-cis-Golgi after knockdown of *baiser* (δ) or *logjam* (γ).

## Discussion

**Figure 10.**
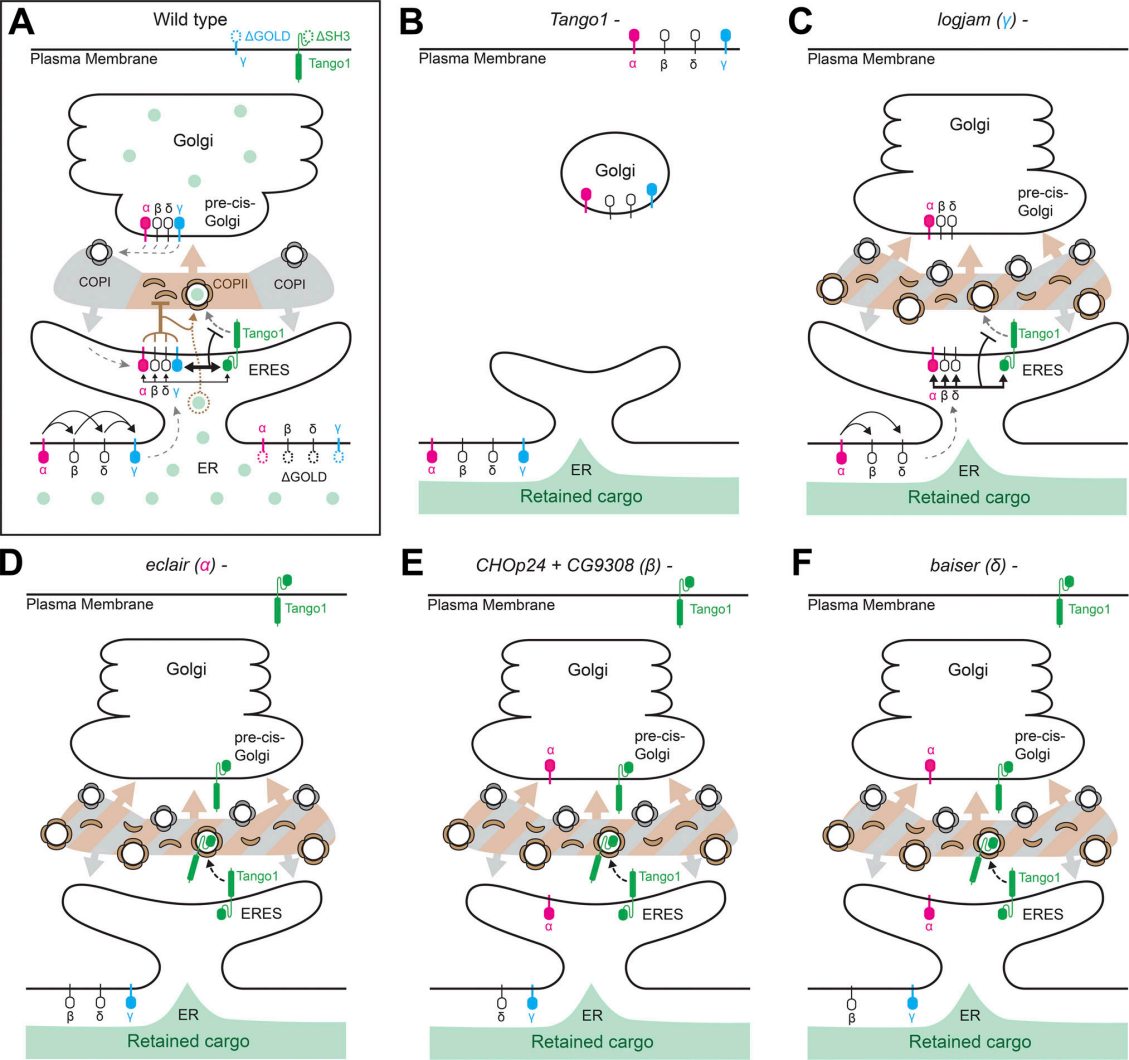
**Tango1–p24 interplay at the ER–Golgi interface. (A–F)** Schematic models depicting localization, interactions, and roles of Tango1 and p24 proteins of the α-, β-, γ-, and δ-p24 subfamilies in wild type (A) and in conditions where Tango1 (B), γ- (C), α- (D), β- (E), or δ-p24 (F) proteins are absent. Concentration of p24 proteins from ER to ERES in wild type (A) follows an α→βδ→γ hierarchy of mutual requirements, possibly reflecting the assembly sequence of a heterotetramer. In this hierarchy, the concentration of β- and δ- require the presence first of α-p24 (D) and each other (E and F), while γ-p24 requires all other three subfamilies. Once complexed at ERES, p24 proteins start cycling between ERES and pre-cis-Golgi transported by COPII (ER-to-Golgi) and COPI (Golgi-to-ER) vesicles (A). Interaction between the p24 GOLD domain (preferentially that of γ-p24) and the SH3 domain of Tango1 aids their concentration at the ER–Golgi interface (A). In the absence of Tango1 (B), uncoupling ERES from Golgi (Liu et al., 2017), p24 proteins are found in both ER and plasma membrane. Conversely, localization of Tango1 at ERES is dependent on p24 proteins, as in the absence of α- (D), β- (E), or δ-p24 (F), but not terminal γ-p24 (C), Tango1 leaves ERES and is trafficked forward to the plasma membrane. Apart from their effects on Tango1, p24 proteins of all four subfamilies are required for efficient general secretion, as in their absence all cargoes we examined were retained in the ER (C–F). This is accompanied by an increase in COPII concentration, excess vesicle budding, and expansion of the central COPII zone at ERES, all evidence of a negative role of p24 proteins on the COPII machinery. To reconcile secretory defects with increased COPII activity, we propose that p24 proteins act as concentrating receptors and ERES stabilizers, binding a wide range of cargoes and other proteins like Tango1 to help their concentration at ERES while retarding their traffic forward.

In this study, we conducted a systematic characterization of p24 proteins in *Drosophila*, their role in the secretory pathway, and the requirements for their localization. Our imaging of α-, β-, γ-, and δ-p24 subfamily proteins showed that they concentrate between the ERES and pre-cis-Golgi, consistent with constant cycling between ER and Golgi. To maintain their localization, besides interactions among different p24 subfamilies (see below), ERES protein Tango1 is required. In the absence of Tango1, p24 proteins fail to concentrate at the ER–Golgi interface and a fraction appears at the plasma membrane. Our further investigation of this relation revealed that Tango1 and p24 proteins physically interact, and that this interaction, in the case at least of γ-p24 Logjam, involves the p24 GOLD domain and the SH3 domain of Tango1, both located in the ER lumen. Other methods beyond coimmunoprecipitation, such as surface plasmon resonance, would be needed to confirm that this interaction is direct in molecular terms. Interestingly, the relation between p24 proteins and Tango1 is mutual as Tango1 requires p24 presence as well to localize to ERES. Loss of p24 proteins α-, β-, or δ-p24 results in Tango1 mislocalization to the plasma membrane, whereas our results indicate that γ-p24 loss can be compensated by the other three. Overall, our results demonstrate that Tango1–p24 interplay is fundamental for maintaining a stable ER–Golgi interface (Fig. 10).

To maintain the localization of p24 proteins, in addition, we were able to determine mutual requirements among the different p24 protein subfamilies. These requirements follow an α→βδ→γ hierarchy in which α-p24 is capable of localizing to the ER–Golgi interface independently, β- and δ-p24 depend on the presence of α-p24 and of each other, and, lastly, γ-p24 needs the presence of all others. What determines these differential behaviors needs further investigation since p24 proteins of all four subfamilies are very similar in sequence and organization. Due also to this similarity, it has been unclear whether p24 proteins play distinct roles or have overlapping functions. In this regard, our study strongly supports that p24 proteins of different subfamilies function non-redundantly as part of heterotetrameric complexes formed in the ER.

Except for α-p24, localizing correctly by itself, complexing would be required for β-, γ-, or δ-p24 entry into ERES or for initial concentration there since they are found throughout the ER when mislocalized. In turn, γ-p24, for which the loss does not affect localization of any of the others, would be the last one to be incorporated into the putative heterotetramer. There is ample support for the existence of p24 heterotetramers in preceding studies (Füllekrug et al., 1999; Marzioch et al., 1999; Fujita et al., 2011; Hirata et al., 2013). Furthermore, the central position of β- and δ-p24 is consistent with previous characterizations of human p24 heterocomplexes (Nagae et al., 2016). It is also suggestive, in that same vein, that only β- and δ-p24 subfamilies are present in plants (Chen et al., 2012; Montesinos et al., 2012).

Previous studies indicate that p24 mutual interactions for assembly and oligomerization take place through their lumenal coiled-coil regions (Ciufo and Boyd, 2000; Emery et al., 2000; Jenne et al., 2002; Chen et al., 2012). However, involvement of their GOLD domains has received support as well (Nagae et al., 2016). While we cannot rule out that GOLD domains are involved in mutual p24 interactions, our results suggest that mislocalization of p24 GOLD deletion mutants is due instead to failed interaction with Tango1. The key result indicating this is that Eclair.ΔGOLD (α) mislocalizes, same as full-length Eclair upon Tango1 knockdown, while Eclair in the absence of other p24 subfamilies does not. It is still possible, however, that both coiled coils and GOLD domains are involved in mutual p24 interactions. Furthermore, if βδ interaction depended on GOLD domains but α→βδ and βδ→γ depended on coiled coils, this would still fit our results. Therefore, further structure-function analysis, including deletion of coiled-coil regions, would be needed to fully understand p24 complex formation.

Consistent as well with the localization hierarchy we deduced, our phenotypic analysis demonstrates that p24 proteins are not functionally redundant across subfamilies, as members of all four subfamilies are required for efficient secretion, a further indication of a single complex performing a common function. We cannot rule out, however, that monomers or complexes different from heterotetramers, such as heterodimers or homodimers, have final functions different from mere assembly intermediates. In fact, our results also argue that a partial complex lacking terminal γ-p24 retains some functionality. Indeed, loss of γ-p24 (Logjam) displays no Tango1 mislocalization. This is despite the fact that Logjam presents the strongest interaction with Tango1, showing that α-, β-, and δ-p24 can cover for its loss. Therefore, while p24 proteins of the four subfamilies play non-redundant roles in the localization hierarchy, they may be able to bind redundantly through their GOLD domains Tango1 (and perhaps other proteins), albeit with different affinities. Apart from their terminal position in the localization hierarchy, γ-p24s differ from the three other subfamilies in other respects: (1) γ-p24 cytoplasmic tails are missing a COPI binding motif present in α-, β-, and δ-p24 members, which could explain the fact that Logjam.ΔGOLD appears on the plasma membrane, while GOLD deletion mutants for the three other subfamilies do not; (2) γ-p24 GOLD domains present an outward-facing, negatively charged surface, in contrast with a more positively charged surface of α-, β-, and δ-p24 GOLD domains (Mota et al., 2022); and (3) γ-p24 is usually the most diversified subfamily (four out of nine genes in *Drosophila*, five out of 10 in humans).

A scenario emerges from our findings here in which multiple mechanisms act on p24 proteins and Tango1 to maintain their localizations at the ER–Golgi interface. We propose that the balance of these forces results in a dynamic equilibrium that maintains a stable interface, ensuring its correct organization and efficient protein transport through it. For p24 proteins, forces influencing their localization are (1) GOLD–SH3 interaction with Tango1, as in the absence of Tango1 all p24 proteins fail to concentrate at ERES and are instead found in both ER and plasma membrane; (2) interactions with p24 proteins of other subfamilies (except for α-p24) facilitates ERES concentration and may be required for their forward ER–Golgi transport since p24 proteins mislocalized due to the absence of others (β, γ, and δ in the absence of α; δ and γ in absence of β; and β and γ in absence of δ) do not appear on the plasma membrane; and (3) retrograde transport that recycles p24 proteins from Golgi back to ERES, consistent with their binding to COPI, except for γ-p24, lacking the COPI binding motif (Dominguez et al., 1998; Fiedler et al., 1996). Meanwhile, Tango1 concentration at ERES would depend on (a) Tango1–Tango1 self-interaction and interaction with other proteins through its cytoplasmic domains, consistent with our finding that the cytoplasmic part of the protein is sufficient to localize Tango1 to ERES (Liu et al., 2017); (b) SH3–GOLD interaction with p24 proteins, which impedes exit of Tango1 from ERES.

It is worth pointing out that the cytoplasmic part of Tango1, besides exhibiting correct localization to ERES by itself, was able to alleviate secretory block and rescue viability when expressed in fat body cells in which endogenous, full-length Tango1 had been knocked down (Liu et al., 2017). From our results here, showing the involvement of the Tango1 lumenal SH3 domain in the correct localization of p24 proteins, it follows that Tango1 cytoplasmic-only Tango1 may not restore p24 concentration, and, therefore, such rescue could only be partial. A second, interesting possibility, however, is that expression of cytoplasmic Tango1 does not just have a rescuing effect but also one of enhancing secretion to the point of overcoming the negative effect on secretion of p24 functional loss.

In addition to the above-mentioned localization mechanisms, it has been reported that KDEL receptor (KdelR)–dependent Golgi-to-ERES recycling acts on both p24 proteins (Majoul et al., 2001) and Tango1 (Yuan et al., 2018). When we knocked down *Drosophila* KdelR, however, we did not see an effect on their localization. This may be due to qualitative differences across organisms or, alternatively, reflect that the influence of KdelR-dependent recycling of these proteins in *Drosophila* is not significant, its loss buffered by the added influence of other localization inputs. Nonetheless, our SIM imaging and previous visualization of Tango1 through APEX (engineered ascorbate peroxidase)-TEM (Liu et al., 2017; Yang et al., 2021) seem to confirm that indeed concentration of *Drosophila* Tango1 at ERES does not result from its constant recycling from the Golgi but from a lack of forward transport from ERES.

Our functional characterization of p24 proteins, in addition, importantly uncovered a broad role for them in secretion. p24 proteins are widely regarded as specific transport receptors for ER–Golgi traffic of particular cargoes such as GPI-anchored proteins (Muñiz et al., 2000; Schimmöller et al., 1995), WNT ligands (Port et al., 2011; Zang et al., 2015), insulin (Zhang and Volchuk, 2010), or interleukin-1 (Zhang et al., 2020). In stark contrast, we show that knockdown of p24 proteins in fat body adipocytes causes intracellular retention of all cargoes we examined. Along with general secretion defects, we observed increased presence of COPII coatomer Sec13 and GTPase Sar1 at ERES, expanding their central COPII zone. At the same time, FIB-SEM analysis revealed an excessive number of ERES–Golgi vesicles compared with the wild type. These defects were also seen after γ-p24 knockdown, a condition in which Tango1 localization at ERES appears undisturbed. Therefore, general secretion impairments are likely caused directly by the loss of p24 proteins, rather than through Tango1 escape. Although the amount of COPII vesicles increases, the fact that general secretion is defective indicates that those vesicles do not mediate efficient transport. Our data, therefore, strongly support that p24 proteins negatively regulate the COPII machinery to prevent unproductive vesicle budding. Negative regulation of COPII vesicle biogenesis by p24 proteins agrees well with the fact that p24 mutations suppress Sec13 mutants in yeast (D’Arcangelo et al., 2015; Elrod-Erickson and Kaiser, 1996; Marzioch et al., 1999).

Expansion of the COPII zone and excess budding could be interpreted as a lax ER retention phenotype due to increased non-selective bulk flow (Gomez-Navarro et al., 2020; Lopez et al., 2020; Ma et al., 2017). p24 proteins, for instance, could take up space inside COPII vesicles, competing as decoys with non-cargo proteins for loading. However, arguing against this interpretation, cargoes retained in fat body adipocytes upon p24 loss included secreted GFP (GFP coupled to a secretion signal peptide), which should exit the ER through bulk secretion. Furthermore, for increased bulk secretion to result in more vesicles, as observed, bulk cargoes should be able to recruit COPII and promote their own transport. An alternative explanation that would better fit our data is that p24 proteins function as concentrating receptors: through their lumenal GOLD domains, p24 proteins perhaps bind a broad range of cargoes and are required to concentrate them at ERES, same as they are required for concentration of Tango1; meanwhile, through their cytoplasmic tails, p24 proteins could interact negatively with COPII to delay budding events and allow cargo loading. Interestingly, both Tango1 and p24 proteins bind COPII, and it has been proposed that Tango1 delays vesicle budding or excision to aid transport of cargoes bound to its SH3 domain (Raote et al., 2018; Saito et al., 2009). Therefore, Tango1 and p24 proteins could have cooperative retardatory effects. In summary, we hypothesize that Tango1 and p24 proteins may both function as concentration receptors and ERES stabilizers (rather than as transport receptors or transport decoys) by binding an ample spectrum of cargoes and other proteins in the ER lumen through their SH3 and GOLD domains, helping their concentration while retarding their exit from ERES. Additionally, future support for this model could come from the analysis of p24 mutants lacking COPII binding motifs and proteomic analysis of the different p24 GOLD domain interactomes.

Our study, finally, adds support to a central role of Tango1 in defining and maintaining ERES. Here, we proved a requirement of Tango1 in maintaining localization of p24 proteins through an SH3-GOLD domain interaction. From previous studies, Tango1 is known to interact through its cytoplasmic part with COPII (Saito et al., 2009), Syntaxin 18 (Nogueira et al., 2014), Rab1, Grasp65 (Liu et al., 2017), Sec16, and Sec12 (Maeda et al., 2017). Numerous proteins of the ER–Golgi interface, therefore, are coordinately bound by Tango1. Moreover, ERES reassembly after mitosis has been shown to depend on Tango1 (Maeda et al., 2020). Further supporting a structural role for Tango1 in the maintenance of the ER–Golgi interface, the loss of *Drosophila* Tango1 reduced the size of ERES and uncoupled them from Golgi, while overexpression of Tango1 created larger ERES (Liu et al., 2017; Yang et al., 2021). In light of all this evidence, we propose that Tango1 ensures the building of a more stable ER–Golgi interface in animal cells through its multiple interactions, including lumenal binding to p24 proteins.

## Materials and methods

### *Drosophila* husbandry

Standard fly husbandry techniques and genetic methodologies, including balancers and dominant markers, were used to assess segregation of transgenes in the progeny of crosses, construct intermediate lines, and obtain flies of the required genotypes for each experiment. Detailed genotypes in each experiment are provided in Table S1. Flies were cultured at 25°C in all experiments. The GAL4-UAS binary expression system was used to drive expression of UAS transgenes under temporal and spatial control of fat body GAL4 driver *Cg-GAL4* (second chromosome) or *BM-40-SPARC-GAL4* (third chromosome). Stable insertion of transgenic UAS constructs was achieved through standard P-element transposon transgenesis at Tsinghua Fly Center. Endogenous tagging was achieved through CRISPR/Cas9-assisted insertion (Peng et al., 2019) at Tsinghua Fly Center. The following strains were used:

*w*^*1118*^ (BDSC:3605)
*w*; *Cg-GAL4* (BDSC:7011)
*w*; *BM-40-SPARC-GAL4 UAS-Dcr2/TM6B* (Liu et al., 2017)
*w*; *UAS-eclair.RNAi*^*101388/KK*^ (VDRC:101388)
*y sc v*; *UAS-logjam.RNAi*^*HMS06058*^ (THFC:TH04039.N)
*y sc v*; *UAS-CHOp24.RNAi*^*HMC05582*^ (THFC:TH04235.N)
*y sc v*; *UAS-p24-1.RNAi*^*HMC04970*^ (THFC:TH04238.N)
*y sc v*; *UAS-p24-2.RNAi*^*HMS02005*^ (THFC:THU4082)
*w*; *UAS-CG9308.RNAi*^*6606/GD*^ (VDRC:6606)
*w*; *UAS-CG31787.RNAi*^*6372/GD*^ (VDRC:6372)
*y sc v sev*; *UAS-opossum.RNAi*^*HMC02679*^ (BDSC:43280)
*w*; *UAS-baiser.RNAi*^*100612/KK*^ (VDRC:100612)
*y w*; *vkg*^*G454*^*-GFP/CyO* (DGRC:11069)
*w*; *UAS-myr-RFP* (BDSC:7118)
*y w*; *Rfabg-sGFP*^*fTRG.900*^ (VDRC:318255)
*w*; *UAS-GFP-GPI/*(*CyO*) (Greco et al., 2001)
*w*; *UAS-mCD8-GFP/CyO* (BDSC:5137)
*w*; *UAS-secr-GFP* (Pfeiffer et al., 2000)
*w*; *UAS-GFP-KDEL* (BDSC:9898)
*y sc v sev*; *UAS-KdelR.RNAi*^*HMC05779*^ (BDSC:64906)
*w*; *UAS-ManII.TagRFP* (BDSC:65249)
*w*; *UAS-GFP-Eclair* (This study)
*w*; *UAS-GFP-CHOp24* (This study)
*w*; *UAS-GFP-Logjam* (This study)
*w*; *UAS-GFP-Baiser* (This study)
*w*; *[mCherry-APEX-Flag]Logjam* (This study)
*w*; *UAS-GalT-TagRFP*; *TM2/TM6B*, *Tb* (BDSC:65251)
*w*; *UAS-ManII-EGFP*; *TM2/TM6B*, *Tb* (BDSC:65248)
*w Gmap*^*KM102*^*-GFP* (DGRC:109702)
*w*; *Grasp65[GFP-APEX-FLAG]* (This study)
*w*; *Sec13[GFP-APEX-FLAG]* (This study)
*y sc v sev*; *UAS-Grasp65.RNAi*^*HMC05584*^ (BDSC:64565)
*w*; *UAS-Ergic53.RNAi*^*108445/KK*^ (VDRC:108445)
*w*; *UAS-Tango1.RNAi*^*NIG11098R*^*/TM6B* (NIG:11098R)
*w*; *UAS-Tango1-GFP* (Liu et al., 2017)
*w*; *UAS-Tango1.*Δ*SH3-GFP* (This study)
*w*; *UAS-GFP-Eclair.*Δ*GOLD* (This study)
*w*; *UAS-GFP-CHOp24.*Δ*GOLD* (This study)
*w*; *UAS-GFP-Logjam.*Δ*GOLD* (This study)
*w*; *UAS-GFP-Baiser.*Δ*GOLD* (This study)
*w*; *UAS-Sar1-GFP-APEX* (Yang et al., 2021)
*y w*; *Kr*^*If-1*^*/CyO*; *UAS-γCOP-mRFP* (BDSC:29714)

### Transgenic constructs

#### UAS-GFP-Eclair, UAS-GFP-CHOp24, UAS-GFP-Logjam, and UAS-GFP-Baiser

To produce each construct, the coding sequence of each gene was amplified from whole larva cDNA using the PrimeScript RT-PCR Kit (cat #RR014-A; Takara). The amplified sequence was then purified through gel extraction (cat #D2111-03; Magen HiPure Gel Pure DNA Mini kit). Flanking att sequences were added through another round of PCR (cat #R011; Takara) and purified. The resulting products were then recombined into pDONR221 (cat #12536017; Thermo Fisher Scientific) through a Gateway BP reaction with Gateway BP Clonase II Enzyme Mix (cat #11789020; Thermo Fisher Scientific) to produce pDONR221-p24 entry clones. From there, p24 sequences were transferred into modified Gateway destination vector pTSGW (UASt-Signal peptide of Tango1-GFP-Gateway cassette) (Yang et al., 2021) through Gateway LR recombination using LR Clonase II Plus enzyme (cat #12538120; Thermo Fisher Scientific) to obtain the desired plasmids.

Primers used were as follows: Eclair-F, Eclair-R, att-Eclair-F, and att-Eclair-R; CHOp24-F, CHOp24-R, att-CHOp24-F, and att-CHOp24-R; Logjam-F, Logjam-R, att-Logjam-F, and att-Logjam-R; and Baiser-F, Baiser-R, att-Baiser-F, and att-Baiser-R. Primer sequences are listed in Table S2.

#### UAS-GFP-Eclair.ΔGOLD, UAS-GFP-CHOp24.ΔGOLD, UAS-GFP-Logjam.ΔGOLD, and UAS-GFP-Baiser.ΔGOLD

GOLD domain deletions were generated from the above full-length p24 gene sequences with primers EclairΔGOLD-F and EclairΔGOLD-R; CHOp24ΔGOLD-F and CHOp24ΔGOLD-R; LogjamΔGOLD-F and LogjamΔGOLD-R; and BaiserΔGOLD-F and BaiserΔGOLD-R. Flanking att sequences were then added through another round of PCR with primers att-Eclair-F and att-Eclair-R; att-CHOp24-R and att-CHOp24-R; att-Logjam-F and att-Logjam-R; and att-Baiser-F and att-Baiser-R. Primer sequences are listed in Table S2. The resulting products were recombined into pDONR221 and transferred into pTSGW as described above.

#### UAS-Tango1.ΔSH3-GFP

pDONR-Tango1ΔSH3 was generated through deletion PCR from pDONR-Tango1 (Liu et al., 2017) with primers Tango1ΔSH3-F and Tango1ΔSH3-R, and from there transferred into pTWG (UASt-Gateway cassette-GFP, *Drosophila* Carnegie Vector Collection) through Gateway LR recombination using LR Clonase II Plus enzyme. Primer sequences are listed in Table S2.

### CRISPR knock-in of [mCherry-APEX-FLAG]Logjam, Grasp65[GFP-APEX-FLAG], and Sec13[GFP-APEX-FLAG]

For knock-in of each gene, three plasmids were used: pU57-Donor-(gene of interest), pU6b-sgRNA-(gene of interest), and universal pU6b-sgRNA1. pU57-Donor consists of universal sgRNA1 sequence, 200 bp upstream sequence from the target site, tagging sequence, linker, 200 bp downstream sequence from the target site, and the sgRNA1 sequence. The target site of Logjam was right after its signal peptide sequence, while for Grasp65 and Sec13 it was C-terminal before their stop codons. pU57-Donor-Logjam, pU57-Donor-Grasp65, and pU57-Donor-Sec13 were synthesized by TsingKe Biotechnology Co., Ltd.

For preparing pU6b-sgRNA for each gene, sgRNAs were selected on the website http://targetfinder.flycrispr.neuro.brown.edu, and oligos were synthesized with TTCG and AAAC added at 5′ end of forward and reverse chain, respectively. Then, sgRNA oligos were annealed and phosphorylated with T4 PNK (cat #M0201; NEW ENGLAND BioLabs) and T4 ligase buffer (cat #M0202V; NEW ENGLAND BioLabs). Next, sgRNA oligos were cloned into pU6b through a BbSI restriction enzyme site (cat #R0539V; NEW ENGLAND BioLabs) and T4 DNA ligation (cat #M0202V; NEW ENGLAND BioLabs) to obtain pU6b-sgRNA-Logjam, pU6b-sgRNA-Grasp65, and pU6b-sgRNA-Sec13. pU6b-sgRNA1 was prepared in the same way with a pair of universal sgRNA1 oligos.

The mixture of pU57-Donor-(gene of interest), pU6b-sgRNA-(gene of interest), and pU6b-sgRNA1 was injected into *y sc v*; *nos-Cas9* embryos at Tsinghua Fly Center. Selected transgenic flies were all homozygous viable. Knock-in sites were validated by genome DNA sequencing. The detailed sequence of each component in pU57-Donor and the sequence of each sgRNA used are listed in Table S2.

### Quantitative real-time PCR

Total RNA was extracted from fat body tissue using TRIzol reagent (cat #15596026; Thermo Fisher Scientific) and used as a template for cDNA synthesis using ABScript III Reverse Transcriptase (cat #RK20408; Abclonal). RT-PCR reactions were performed using SYBR Green Supermix (cat #1725120; Bio-Rad) in a CFX96 Real-Time PCR system (Bio-Rad). Expression values were normalized to *Rp49* transcript levels. The relative expression level with respect to the wild-type control was calculated by the Delta–Delta Ct method. Three separate biological replicates were performed for each experiment, each with three technical replicates. Primers used were as follows: eclair-rt-F, eclair-rt-R; p24-2-rt-F, p24-2-rt-R; CHOp24-rt-F, CHOp24-rt-R; CG9308-rt-F, CG9308-rt-R; logjam-rt-F, logjam-rt-R; opossum-rt-F, opossum-rt-R; CG31787-rt-F, CG31787-rt-R; p24-1-rt-F, p24-1-rt-R; baiser-rt-F, baiser-rt-R; and Rp49-rt-F, Rp49-rt-R. Primer sequences are listed in Table S2.

### Confocal and 3D-SIM superresolution imaging

L3 larvae were predissected in PBS by turning them inside out with fine-tip forceps, fixed in PBS containing 4% PFA (paraformaldehyde, cat #80096692; Sinopharm Chemical Reagent) for 15 min, washed in PBS for 3 × 10 min, dissected from the carcass, and mounted on a glass slide with a drop of DAPI-Vectashield (cat #H-1200; Vector Laboratories). Confocal images were acquired with a ZEISS LSM780 microscope equipped with a 63× oil Plan-Apochromat objective (NA 1.4) and a 100× oil Plan-Apochromat objective (NA 1.4).

SIM image stacks (z-steps of 0.24 μm) were acquired with a Nikon A1 N-SIM STORM microscope equipped with a CFI Apo SR TIRF 100× oil (NA 1.49) objective and an Andor Technology EMCCD camera (iXON DU-897 X-9255). Laser lines at 488, 560, and 640 nm were used for excitation. SIM image reconstructions were performed with NIS-Elements software (Nikon). Images are maximum intensity projections of three to five sections.

### Immunohistochemistry

Antibody staining was performed using standard procedures for larval tissues. Briefly, larvae were predissected in PBS; fixed in PBS containing 4% PFA for 15 min; washed in PBS for 3 × 10 min; blocked in PBT-BSA (PBS containing 0.1% Triton X-100 detergent [cat #T8787; Sigma-Aldrich], 1% BSA [cat #201903A28; Zhongkekeao], and 250 mM NaCl); and incubated overnight with primary antibody in PBT-BSA at 4°C on a rotator. The next day, tissues were washed in PBT-BSA for 3 × 20 min, incubated for 2 h with secondary antibody in PBT-BSA at room temperature, and washed in PBT-BSA for 3 × 20 min and then PBS for 3 × 10 min. Fat body tissues were finally dissected and mounted on a glass slide with DAPI-Vectashield. The primary antibody guinea pig anti-Tango1 (Lerner et al., 2013) (1:1,000) was used. Secondary antibodies were goat anti-guinea pig IgG (1: 200, Alexa Fluor 488 conjugated, cat #106545003; Jackson ImmunoResearch; 1:200, Alexa Fluor 647 conjugated, Jackson ImmunoResearch, cat #106605003; Jackson ImmunoResearch).

### Immunoprecipitation

L3 fat body from 200 larvae was collected and homogenized on ice using an electric pellet pestle and a lysis buffer containing 10 mM Tris-HCl (pH = 7.5), 0.5 mM EDTA, 150 mM NaCl, 0.5% NP-40 (cat #1221A21; Leagene), and 1× protease inhibitor (cat #P1005; Beyotime). Samples were then cleared through centrifuging for 15 min at 20,000 *g* and 4°C. Protein concentration of lysates was quantified using a BCA kit (cat #23227; Thermo Fisher Scientific) with a NanoDrop 2000C. GFP and Flag immunoprecipitation experiments were then conducted according to the manufacturer’s instructions.

For GFP immunoprecipitation, GFP-Trap agarose beads (cat #GT10; ChromoTek) were first washed with IP buffer (10 mM Tris-HCl, 150 mM NaCl, 0.5 mM EDTA, pH = 7.5) for 3 × 1 min and collected through centrifuging for 1 min at 5,000 *g*, 4°C. Samples were incubated with pre-washed GFP agarose beads and rotated overnight at 4°C. Then, beads were collected through centrifuging for 1 min at 5,000 *g*, 4°C. Next, beads were washed 5 × 1 min with IP buffer and collected by centrifuging for 1 min at 5,000 *g*, 4°C. Finally, 2 × SDS-PAGE buffer (120 mM Tris-HCl, 4% SDS, 20% glycerol, 0.5% bromophenol blue, and 5% β-mercaptoethanol) was added to samples and proteins were eluted from GFP agarose beads through boiling at 95°C for 10 min and cooled down on ice.

For Flag immunoprecipitation, anti-Flag magnetic beads (cat #M8823; Sigma-Aldrich) were washed using 1 × TBS buffer (50 mM Tris-HCl, 150 mM NaCl, pH = 7.5) three times and collected by a magnetic rack. Samples were incubated with prewashed anti-Flag magnetic beads and rotated overnight at 4°C. Then, beads were collected and washed for 5 × 1 min with 1 × TBS buffer. Proteins were eluted from Flag magnetic beads through adding five packages of beads with a gel volume of 150 ng/μl 3 × Flag peptide (cat #4799; Sigma-Aldrich) in 1 × TBS buffer and incubating in a rotator for 1 h at 4°C. Then, the supernatant was collected, added with 5 × SDS protein loading buffer (250 mM Tris-HCl, 10% SDS, 50% glycerol, 0.5% bromophenol blue, and 5% β-mercaptoethanol), boiled at 95°C for 10 min, and cooled down on ice.

### Western blotting

Protein lysates added with 5 × SDS protein loading buffer were boiled at 95°C for 10 min for reducing. Then, samples were loaded in a 4–20% SDS-PAGE gradient gel (cat #P0057A; Beyotime) or 15% SDS-PAGE gel (cat #P0055B; Beyotime) and separated by electrophoresis in 1 × SDS-PAGE running buffer (cat #P0014A; Beyotime) at 120 V. Proteins were then transferred to a polyvinylidene difluoride membrane (cat #1620177; Bio-rad) for 70 min at 300 mA and blocked in 5% skim milk in TBST (50 mM Tris-HCl, 150 mM NaCl, 0.5% Tween-20, pH = 8) for 1 h at room temperature. Primary antibodies were diluted in blocking solution and incubated overnight at 4°C on a rotator. The next day, membranes were washed 3 × 10 min with TBST, incubated with secondary antibodies diluted in TBST for 1 h at room temperature on a rotator, washed 3 × 10 min with TBST, and exposed with an ECL kit (cat #1705061; Bio-rad) on x-ray films (cat #JPKD-5; Kodak). Primary antibodies used were: anti-Tango1 (Lerner et al., 2013) (1:5,000), anti-GFP (1:3,000, cat #1814460001; Roche), and anti-Flag (1:3,000, cat #F1084; Sigma-Aldrich). Secondary antibodies used were: Goat anti-mouse IgG-HRP (1:10,000, cat #M21001L; Abmart) and Goat anti-Guinea pig IgG-HRP (1:10,000, cat #ab6908; Abcam).

### FIB-SEM imaging

FIB-SEM imaging was performed as previously described (Yang et al., 2021). Resin blocks were trimmed to expose tissues and then fixed onto a 45/90° screw-type holder. The samples were subsequently coated with a layer of gold using a HITACHI E-1010 ion sputter coater for 120 s. FIB-SEM imaging was performed using an FEI Helios NanoLab G3 dual-beam microscope system equipped with Everhart-Thornley detector, TLD (through-lens detector), and ICD (in-column detector) cameras (Thermo Fisher Scientific). During the milling of slices, an ion beam current of 0.43 nA at a 30 kV acceleration voltage was employed, with each milling step set at 20 nm. For the SEM imaging, the following parameters were utilized: a beam current of 0.4 nA, an acceleration voltage of 2 kV, a working distance of 2 mm, a dwell time of 8 µs, a pixel size of 3–4 nm, and a pixel count of 4,096 × 3,536. TLD and ICD cameras collected backscattered signals for imaging. The imaging software used was AutoSlice and View G3 1.7.2 (FEI).

Images obtained from FIB-SEM were imported into Dragonfly (Object Research Systems). The Dragonfly Image Loader was utilized to import the images and the SSD method in the Slide Registration panel was employed to align them. For segmentation, various regions of interest (ROIs) were created using the ROI tools panel. Each organelle or membrane component was manually segmented as an individual ROI using the ROI Painter round brush tool in 2D mode. Once segmentation was completed for each section, the ROIs were exported and saved as object files. These objects were then converted into 3D meshes by using the export box in the ROI Tools panel. The meshes underwent a smoothing process four to six times and were examined in 3D scene mode, appearing as solid and fully opaque objects. For volume measurements, the information panel was used to record values for each object. The diameter of each vesicle, visible in two to four consecutive sections, was measured on its largest xy section in 2D mode using the Ruler tool in the annotation panel.

### Statistical analysis

Fluorescence intensity, fluorescence profiles, and puncta size were calculated using Image J. Fluorescence intensity of plasma membrane regions in a cell was measured by averaging intensity in multiple 1 × 5 μm rectangles covering the whole plasma membrane. Statistical analysis and graphical representations were performed using GraphPad Prism. Data distribution was tested for normality by D’Agostino and Pearson normality test. P values were calculated by one-way ANOVA and Dunnett’s multiple comparisons tests, Brown–Forsythe ANOVA and Dunnett’s multiple comparisons tests, unpaired two-sided *t* test, or Welch’s *t* test. For all analyses, significance was determined at P < 0.05 (*P < 0.05, **P < 0.01, ***P < 0.001, ****P < 0.0001). Statistical details of each experiment are listed in the figure legends.

### Online supplemental material

Fig. S1 shows p24 expression levels in larval fat body cells. Fig. S2 shows Tango1–p24 localizations are mutually dependent. Fig. S3 shows GOLD domain is required for correct p24 localization. Fig. S4 shows that p24 loss increases Sar1 recruitment and enlarges pre-cis-Golgi. Fig. S5 shows FIB-SEM analysis of mutant p24 ERES–Golgi units. Table S1 shows genotype information (Excel file). Table S2 shows DNA sequences (Excel file). SourceData F6 shows uncropped western blot scans from Fig. 6 (PDF file). SourceData F7 shows uncropped western blot scans from Fig. 7 (PDF file).

## Supplementary Material

Table S1 shows genotype information.

Table S2 shows DNA sequences.

SourceData F6 is the source file for Fig. 6.

SourceData F7 is the source file for Fig. 7.

## Data Availability

All data reported in this paper will be shared by the corresponding author (jose.pastorp@umh.es) upon reasonable request.
