# Supplementary figures and images for: p24–Tango1 interactions ensure ER–Golgi interface stability and efficient transport
Source: J Cell Biol. 2024 Mar 12;223(5):e202309045. doi: 10.1083/jcb.202309045 (PMC10932740; doi:10.1083/jcb.202309045)

**Figure 6A**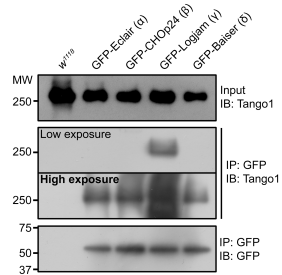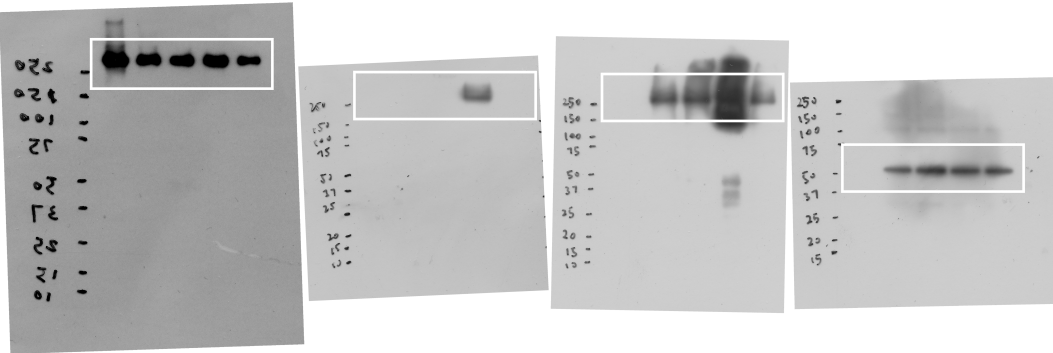**Figure 6B**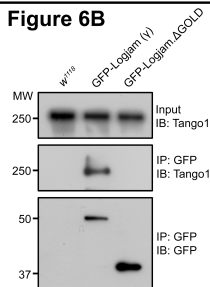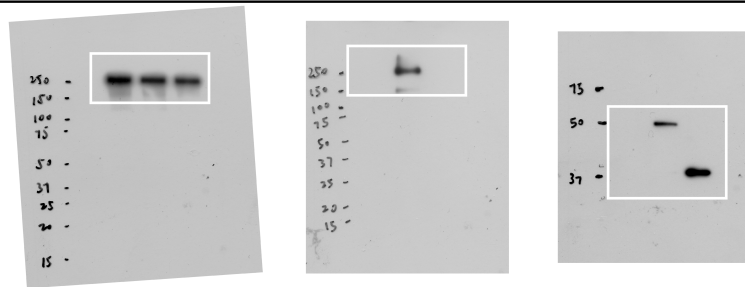**Figure 6C**

[FLAG-mCherry-APEX]  
-Logjam (γ) CRISPR KI

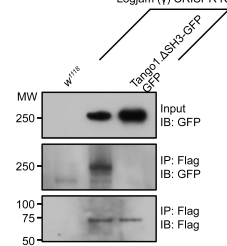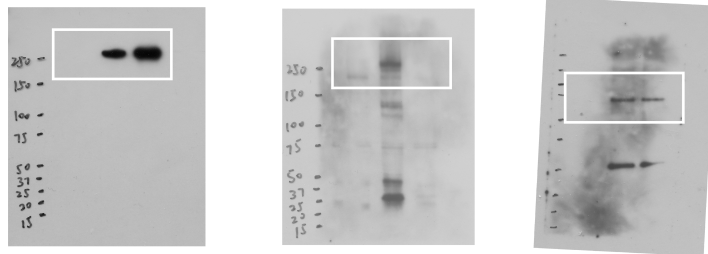

Supplement: SourceData F6 — is the source file for Fig. 6. [file JCB_202309045_SourceDataF6.pdf]

**Figure 7B**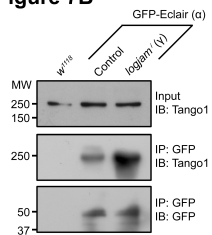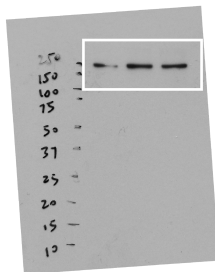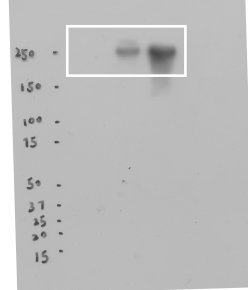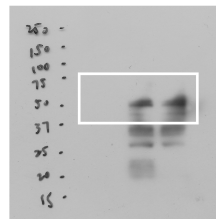**Figure 7C**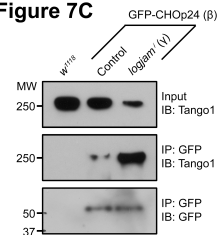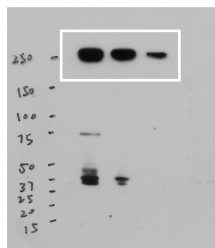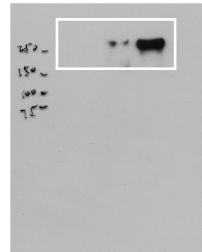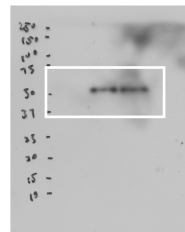**Figure 7D**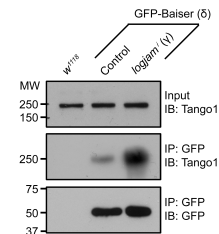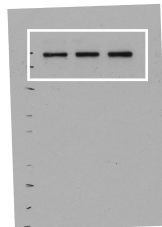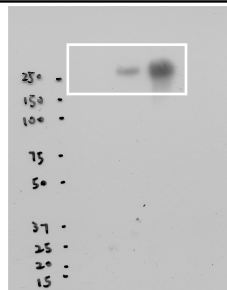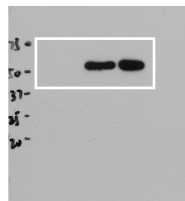

Supplement: SourceData F7 — is the source file for Fig. 7. [file JCB_202309045_SourceDataF7.pdf]
